# Rapid protein evolution, organellar reductions, and invasive intronic elements in the marine aerobic parasite dinoflagellate *Amoebophrya* spp

**DOI:** 10.1186/s12915-020-00927-9

**Published:** 2021-01-06

**Authors:** Sarah Farhat, Phuong Le, Ehsan Kayal, Benjamin Noel, Estelle Bigeard, Erwan Corre, Florian Maumus, Isabelle Florent, Adriana Alberti, Jean-Marc Aury, Tristan Barbeyron, Ruibo Cai, Corinne Da Silva, Benjamin Istace, Karine Labadie, Dominique Marie, Jonathan Mercier, Tsinda Rukwavu, Jeremy Szymczak, Thierry Tonon, Catharina Alves-de-Souza, Pierre Rouzé, Yves Van de Peer, Patrick Wincker, Stephane Rombauts, Betina M. Porcel, Laure Guillou

**Affiliations:** 1 Génomique Métabolique, Genoscope, Institut François Jacob, CEA, CNRS, Univ. Evry, Université Paris-Saclay, 91057 Evry, France; 2 School of Marine and Atmospheric Sciences, Stony Brook University, Stony Brook, New York 11794 USA; 3 Center for Plant Systems Biology, VIB, Ghent, Belgium, & Department of Plant Biotechnology and Bioinformatics, Ghent University, Ghent, Belgium; 4 Sorbonne Université, CNRS, FR2424, Station Biologique de Roscoff, Place Georges Teissier, 29680 Roscoff, France; 5 Sorbonne Université, CNRS, UMR7144 Adaptation et Diversité en Milieu Marin, Ecology of Marine Plankton (ECOMAP), Station Biologique de Roscoff SBR, 29680 Roscoff, France; 6 URGI, INRA, Université Paris-Saclay, 78026 Versailles, France; 7 Unité Molécules de Communication et Adaptation des Microorganismes (MCAM, UMR7245), Muséum national d’Histoire naturelle, CNRS, CP 52, 57 rue Cuvier, 75005 Paris, France; 8 Sorbonne Université, CNRS, UMR 8227, Station Biologique de Roscoff, Place Georges Teissier, 29680 Roscoff, France; 9 Centre for Novel Agricultural Products, Department of Biology, University of York, Heslington, York, YO10 5DD UK; 10 Algal Resources Collection, MARBIONC, Center for Marine Sciences, University of North Carolina Wilmington, 5600 Marvin K. Moss Lane, Wilmington, NC 28409 USA; 11 Department of Biochemistry, Genetics and Microbiology, Pretoria, South Africa

**Keywords:** Non-canonical introns, Introner elements, Genome, Parasite, Dinoflagellate

## Abstract

**Background:**

Dinoflagellates are aquatic protists particularly widespread in the oceans worldwide. Some are responsible for toxic blooms while others live in symbiotic relationships, either as mutualistic symbionts in corals or as parasites infecting other protists and animals. Dinoflagellates harbor atypically large genomes (~ 3 to 250 Gb), with gene organization and gene expression patterns very different from closely related apicomplexan parasites. Here we sequenced and analyzed the genomes of two early-diverging and co-occurring parasitic dinoflagellate *Amoebophrya* strains, to shed light on the emergence of such atypical genomic features, dinoflagellate evolution, and host specialization.

**Results:**

We sequenced, assembled, and annotated high-quality genomes for two *Amoebophrya* strains (A25 and A120), using a combination of Illumina paired-end short-read and Oxford Nanopore Technology (ONT) MinION long-read sequencing approaches. We found a small number of transposable elements, along with short introns and intergenic regions, and a limited number of gene families, together contribute to the compactness of the *Amoebophrya* genomes, a feature potentially linked with parasitism. While the majority of *Amoebophrya* proteins (63.7% of A25 and 59.3% of A120) had no functional assignment, we found many orthologs shared with Dinophyceae. Our analyses revealed a strong tendency for genes encoded by unidirectional clusters and high levels of synteny conservation between the two genomes despite low interspecific protein sequence similarity, suggesting rapid protein evolution. Most strikingly, we identified a large portion of non-canonical introns, including repeated introns, displaying a broad variability of associated splicing motifs never observed among eukaryotes. Those introner elements appear to have the capacity to spread over their respective genomes in a manner similar to transposable elements. Finally, we confirmed the reduction of organelles observed in *Amoebophrya* spp., i.e., loss of the plastid, potential loss of a mitochondrial genome and functions.

**Conclusion:**

These results expand the range of atypical genome features found in basal dinoflagellates and raise questions regarding speciation and the evolutionary mechanisms at play while parastitism was selected for in this particular unicellular lineage.

**Supplementary information:**

The online version contains supplementary material available at 10.1186/s12915-020-00927-9.

## Background

**Fig. 1:**
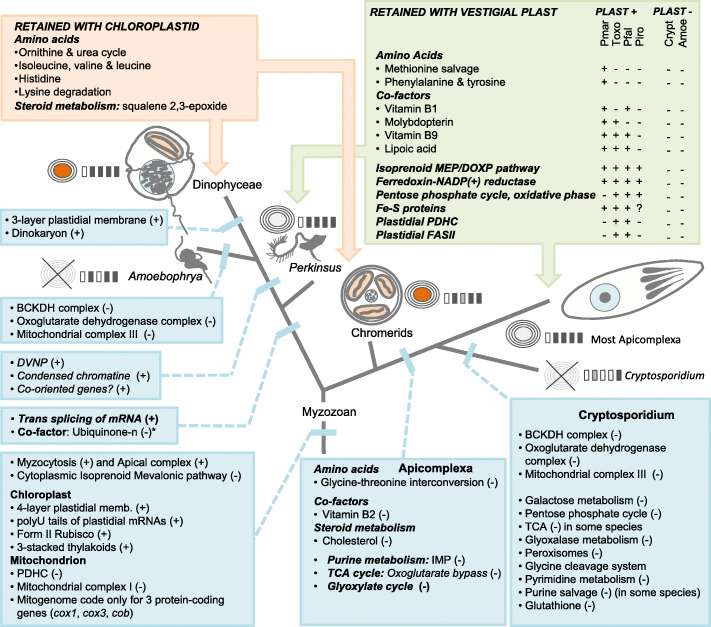
Synthetic view of key functional losses (−) and gains (+) during the evolution of Myzozoa. Blue shaded boxes: metabolic pathways lost or gained during evolution. Orange/green shaded boxes: metabolic pathways potentially lost when a chloroplast or a plast is retained. Amoe: *Amoebophrya* spp., Crypt: *Cryptosporidium* spp., Pfal: *Plasmodium falciparum*, Piro: Piroplasma, Pmar: *Perkinsus marinus*, Toxo: *Toxoplasma gondii,*  : Chloroplast with 3 membranes, : Chloroplast with 4 membranes, : Plastid with 4 membranes (not detected when crossed out), : Illustration of the five complexes of the OXPHOS pathway (white when not detected, dark when detected, gray when dependent on species)

Dinoflagellates (Alveolata, Myzozoa) are single-cell eukaryotes with a wide range of lifestyles. Approximately half of known dinoflagellates are photosynthetic species representing important marine primary producers, with some of them responsible for toxic blooms. Dinoflagellates occur as either free-living organisms or live in symbiosis with other eukaryotes, such as the emblematic Symbiodiniaceae found in corals [1, 2]. Despite differences in habitats and lifestyles, dinoflagellates and their sister groups (including the infamous human malaria parasite *Plasmodium falciparum*) share a common phototrophic myzozoan ancestor that originally acquired its plastid from a red algal endosymbiont [3] or a haptophyte prey [4] (Fig. 1, Fig. S1).

Unlike other alveolates, dinoflagellates posess very large genome sizes (~ 3 to 250 Gb) with 20–270 chromosomes that are relatively gene-rich and nearly permanently packed into condensed liquid-crystalline dinokaryons [5, 6]. Their genetic material is associated with dinoflagellate/viral nucleoproteins (DVNPs) that likely originated from phycodnaviruses [7] and histone-like proteins derived from bacterial HU-like proteins [8]. Gene expression in dinoflagellates involves trans-splicing of messenger RNAs [9] through the addition of a 5′-end dinoflagellate-specific spliced leader (DinoSL) sequence [10, 11], and which is still identifiable in the genomic sequence of presumably retro-transposed transcripts [12]. Furthermore, unusual GC-GA dinucleotide pairs at the 5′-donor splice site of introns [13] and a putative translational (rather than transcriptional) gene regulation mechanism have been suggested in dinoflagellates [14]. Therefore, the exploration of early-diverging dinoflagellate lineages such as the Syndiniales (also known as environmental Marine ALVeolates or MALVs [15]) shall shed light on the emergence of such atypical genomic features.

The Syndiniales *Amoebophrya* spp. are intracellular marine parasites of dinoflagellates, radiolarians, ciliates, and other *Amoebophrya* strains [16, 17]. A single infection by *Amoebophrya*-like parasites can lead to the production of hundreds of infective flagellated propagules called dinospores. While the range of potential hosts varies among strains, those of *Amoebophrya* spp. are generally observed to be highly host-specific in the field and involved in the biological control of dinoflagellate blooms [18–20]. Using a combination of Illumina paired-end short-read and Oxford Nanopore Technology (ONT) MinION long-read sequencing approaches, we sequenced and assembled high-quality genomes for two *Amoebophrya* strains (A25 and A120). Both strains belong to the MALV-II clade 2 lineage (following the nomenclature proposed by Guillou et al. [15]) and share 96.53% of SSU rDNA sequence similarity (Fig. S2). However, recent analyses suggest that these strains belong to two separate cryptic species displaying differential host ranges: A25 (RCC4383) is restricted to the non-toxic autotrophic dinoflagellate *Scrippsiella acuminata*, whereas A120 (RCC4398) can infect a wider range of hosts belonging to at least two dinoflagellates genera (*Scrippsiella* and *Heterocapsa*, Table S1) [21]. We used a comparative genome analysis of these two *Amoebophrya* strains to get insights into the evolution of dinoflagellates and host specialization in *Amoebophrya* spp.

## Results

### Compact genomes among early-diverging dinoflagellates

Genome assemblies of the two *Amoebophrya* sp. strains resulted in cumulative sizes of 116 Mb and 115.5 Mb for A25 and A120, respectively (Table 1, Table S2). These values were consistent with k-mer genome estimates (118.57 and 113.59 Mb in A25 and A120, respectively; Fig. S3) and flow cytometry DNA content measurements (131.60 ± 5.39 and 125.25 ± 5.24 Mb in A25 and A120, respectively). High contiguous genome assemblies were obtained for the *Amoebophrya* strains (scaffold N50 length of 1.08 Mb and 9.24 Mb for A25 and A120 respectively, Table 1). Half of the genome size is contained in 5 scaffolds for A120, thus indicating a close-to-chromosome-level assembly for this strain. The A120 strain also harbors plant-like telomere repeat motifs (TTTAGGG/TTTGGGG) at the end of three scaffolds (numbered 1, 8, and 23), as previously reported in Dinophyceae [22]. Comparatively, the recently published draft genome of the *Amoebophrya* sp. strain AT5 which infects the toxic autotrophic dinoflagellate *Alexandrium catenella* was estimated at 120 Mb by flow cytometry but resulted in a cumulative assembly size of 87.7 Mb (scaffold N50 length of 83.9 kb; Table 1) [23].

**Table 1:** Assembly and annotation metrics of *Amoebophrya* A25, A120, and AT5 genomes, of the Symbiodiniaceae *Breviolum minutum* (Bmin), *Fugacium kawagutii* (Fkav), *S. microadriaticum* (Smic), and for *Perkinsus marinus* (Pmar)

	A25	A120	AT5	Fkav	Bmin	Smic	Pmar
**Assembly**							
Number of scaffolds	557	50	2351	30,040	21,899	9695	17,897
Cumulative size (Mb)	116	115.5	87.7	935	609	808	87
Scaffold N50 / L50	1.082 Mb / 35	9.243 Mb / 5	83.9 kb / 298	381 kb / 772	125 kb / 1448	574 kb / 420	158 kb / 124
Scaffold N90 / L90	423 kb / 106	1.464 Mb / 18	19.6 kb / 1095	109 kb / 2477	31 kb / 5103	146 kb / 1442	1.2 kb / 9284
Scaffold max. size	3.013 Mb	16.512 Mb	537 kb	1.914 Mb	811 kb	3.145 Mb	1.8 Mb
%N	2.27	1.41	2.25	3.4	0.9	7.7	0.64
%GC	47.8	51.2	55.92	45.5	43.5	50.5	47.4
**Genes**							
Number	28,091	26,441	19,925	31,520	32,803	29,728	23,654
Density (genes/Mb)	247.78	232.18	227.2	39.4	68.78	60.8	273.1
Average length (bp)	2965	3482	2782	8836	10,069	9281	1581
Median length (bp)	1890	2442	1803	2039	7899	7255	1038
**Exons**							
Number	117,411	121,327	67,639	150,118	985,369	1,072,528	133,410
Av. length (bp)	475	541	578	256	99	109	177
Median length (bp)	235	265	319	81	53	51	112
Longest (bp)	79,744	44,016	14,772	11,064	14,818	13,755	16,293
Average number of exons / gene	4.18	4.59	3.39	4.07	20.96	21.8	5.64
% GC	51.9%	56.3%	54.7%	52.7%	50.8%	56.9%	50.95%
**Introns**							
Number	81,610	90,882	47,714	113,268	938,355	1,023,342	109,756
% of spliced genes	69.8%	66.9%	71.3%	64.1%	95.4%	98.6%	72.4%
Average length (bp)	345	335	337	893	517	505	124
Median length (bp)	208	247	228	501	297	231	49
Longest (bp)	90,415	35,152	3556	9977	88,176	177,825	11,034
% GC	44%	46.5%	49.4%	44.5%	41.8%	47.1%	43.4%
% of introns with GT-AG splice sites	34.02%	30.41%	99.98%	65.38%	48.23%	0.26	99.3%
% of introns with GC|GA-AG splice sites	0.45%	2.95%	0.02%	25.30%	51.77%	73.95%	0.7%
% of introns with other splices sites	65.53%	66.64%	0%	9.32%	0%	0.05%	0%
**CDS**							
Average coding size (bp)	1337	1773	1962	1041	1916	2375	4839
Genome coverage of coding bases, % in brackets	32.4%	40.6%	44.6%	4.1%	13.1%	14.4%	26.4%
**Gene families**							
Number of genes belonging to families, % in brackets	7074 (25.2)	7428 (28.1)	ND	20,374 (55.3)	25,809 (61.5)	32,796 (66.8)	18,258 (77.2)
Avg. of genes in a family	3.5	3.6	ND	6.7	5.9	7	ND
Max. of genes in a family	171	157	ND	889	703	831	ND
**Annotation**							
Number of proteins with at least one significant match	8360	8690	4366	29,720	13,813	5538	ND
Number of proteins with KO assignation	5774 (21%)	5983 (23%)	2018	14,926 (40%)	10,954 (65%)	3008 (54%)	ND
Number of proteins with BRITE assignation	5774	5856		14,764	10,755	2960	ND
Number of proteins of with an IPR domains	8444	9054	7404	16,895	13,541	4059	ND
Number of proteins with UniProt matches (%)	9101 (32.4)	9404 (35.6)	ND	ND	ND	ND	ND

Gene annotation resulted in the prediction of 28,091 and 26,441 genes in A25 and A120, respectively (Table 1). Predicted gene metrics were similar in terms of number and size to the 23,654 genes described in *Perkinsus marinus*, and predictably higher than the 19,925 genes found in the *Amoebophrya* AT5 strain (Table 1). By comparison, most Symbiodiniaceae (excluding *F. kawagutii*) contain a slightly larger number of genes (~ 29,000–40,000 predicted genes, [24]) which are on average 3–4 times longer in size (Table 1). Similarly, the number of highly conserved tandemly duplicated genes in A25 and A120 was lower (206 and 185, respectively) than those observed in *Symbiodinium microadriaticum* (410), *F. kawagutii* (1004), and *Breviolum minutum* (6278). A low proportion of repetitive elements was observed in both genomes (23.8% and 13.1% in A120 and A25, respectively; Fig. S4), with a majority of them unclassified. Additionally, both genomes contained a diversity of autonomous transposable elements corresponding to several retro-element families, including long terminal repeat (LTR) and non-LTR retrotransposons (Fig. S4).

We identified a truncated DinoSL motif (13 nucleotides, representing 60% of the DinoSL motif; Fig. S5) at the 5′-end of at least 18.5% (A25) and 37.8% (A120) of the transcripts, a similar value found in other published data [13, 25]. These truncated motifs likely derive from a single complete (22 nucleotides) DinoSL-like coding sequence that was also detected in each genome (Fig. S6). In contrast to what has been previously described in other dinoflagellates [13], this gene is not located within a spliceosomal gene cluster in *Amoebophrya* spp.. Interestingly, we found that a large majority of *Amoebophrya* genes were packed into long co-oriented chromosomal regions or “blocks” (98.1% of genes into 587 blocks in A25; 98.5% of genes into 516 blocks in A120; 83% into 1245 blocks in AT5). The average shift of gene orientation (number of time a gene is found in an opposite direction in a sliding window of 10 genes, as described in Shoguchi et al. [26]) was higher in AT5 (0.93) compared to the other two *Amoebophrya* strains (about 0.17 and 0.15 in A25 and A120, respectively), but remained lower than what has been described in most Symbiodiniaceae genomes (2.32 for *S. microadriaticum*, 2.11 for *F. kawagutii*, and 0.64 for *B. minutum*; Fig. S7). This tendency seems to be general to all dinoflagellates [25].

### *Amoebophrya*-specific coding genes

Close to 60% of the KEGG functional units were recovered from the *Amoebophrya* predicted proteomes, with both strains sharing similar metabolic capabilities. However, the majority of *Amoebophrya* proteins (63.7% in A25 and 59.3% in A120) had no functional assignment using KEGG, UniProtKB, or InterPro domain annotations. Based on gene prediction completeness assessment using the Benchmarking Universal Single-Copy Orthologs (BUSCO [27], Eukaryota dataset version 4.0.2), 69.4% and 70.2% of conserved genes were detected in A25 and A120, respectively (this ratio was 65.3% for AT5). Such a result can in part be explained by the relatively high sequence divergence between *Amoebophrya* genes and those of organisms in reference databases. In addition, many intracellular parasites have lost a substantial number of biosynthetic genes.

Using a homology-based approach, we clustered the *Amoebophrya* spp. predicted proteins in the two strains sequenced for this manuscript with those of other parasites belonging to Euglenozoa and Alveolata and those of free-living and symbiotic species (Table 1). This comparison allowed us to group 12,149 genes from A25 and 11,726 genes from A120 into 7320 gene families (OGs), with 3781 *Amoebophrya*-specific OGs shared by both strains containing 5036 and 4665 proteins from A25 and A120, respectively. Among the 3781 *Amoebophyra*-specific OGs shared between both strains, only 1595 proteins from A25 and 1745 from A120 contained recognizable functional domains (Fig. S8). Each strain also contained a substantial proportion of species-specific OGs (genes detected in only one species, Fig. S8): 13,990 in A25 and 12,747 in A120 accounting for 55% (15,407) and 54% (14,255) of total genes for A25 and A120, respectively (Fig. S8), with functional domains assigned to only a small fraction (6% for A25 and 8.5% for A120) of the predicted proteins.

### Genome structure conservation contrasts with protein sequences evolution

**Fig. 2:**
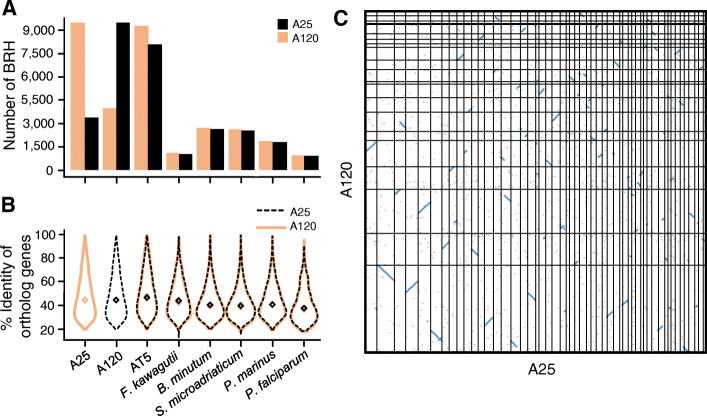
Distribution of the numbers of orthologous and paralogous genes, gene orthology, and synteny in the A25 and A120 genomes. **a** Number of orthologous and paralogous genes defined by Best Reciprocal Hit (BRH) searches between A25 (blue), A120 (yellow), *Amoebophrya* AT5, *P. falciparum*, *P. marinus*, *F. kawagutii*, *S. microadriaticum*, and *B. minutum* predicted proteomes. **b** Violin distribution of the percent identity of orthologous genes defined by best reciprocal hits (BRHs) between *Amoebophrya* A120 (in peach), A25 (in dark), and a selection of other alveolates, including *Amoebophrya* strain AT5 (in brown). Diamonds represent median values for each distribution. **c** Dot-plot of the synteny observed between the longest scaffolds for each of the *Amoebophrya* A25 (*x*-axis, 53 scaffolds) and A120 (*y*-axis, 21 scaffolds) genomes. For each genome, genes are sorted by their rank on the scaffolds. Each dot represents a pair of orthologous genes defined by BRH. Blue lines highlight syntenic regions

The three *Amoebophrya* strains shared only 8118 to 9490 orthologous genes, representing 36–47% of the total number of predicted protein genes in each strain (Fig. 2a). These orthologs shared 48.2–51.2% amino acid sequence identity on average, a level similar to what was observed when comparing each *Amoebophrya* strain with Symbiodiniaceae, the perkinsid *P. marinus* and the apicomplexan *P. falciparum* (Fig. 2b). We estimated a dN/dS below 1 (0.6) on average (Fig. S9), which might suggest the importance of a purifying selection (natural selection suppresses protein changes). About a quarter of orthologous proteins (22%) had a ratio superior to 1; they could be good candidates to investigate divergent selection between the two lineages. However, despite large protein sequences divergences, A25 and A120 genomes exhibited strong synteny conservation with 64% of homologous genes (6908 out of 9490) clustered into 196 collinear syntenic blocks containing 84% (A120) and 80% (A25) of the total number of predicted genes (Fig. 2c). Despite the highly fragmented state of the AT5 genome assembly, we also found a rather high level of synteny conservation of orthologous genes between AT5 and the strains sequenced here (49% with A25 and 57% with A120, Figs. S10-S11).

### Loss of plastids in *Amoebophrya*

We did not find any genetic evidence for plastidial functions in the A25 and A120 genomes. This is illustrated by the absence of (1) genes encoding light-dependent reactions, (2) genes maintained in non-photosynthetic plastids such as *sufB* (a subunit of the Fe-S cluster assembly) and *clpC* (a subunit of the ATP-dependent Clp protease), (3) the plastidial fatty acid synthase type II pathway and enzymes involved in plastidial fatty acid metabolism (e.g., fatty acyl-ACP thiosterases), (4) genes coding for the synthesis of thylakoid membrane lipids (sulfolipids and galactolipids, UDP-sulfoquinovose synthase (SQD1), sulfoquinovosyltransferase (SQD2), monogalactosyldiacylglycerol synthase (MGDGS), and digalactosyldiacylglycerol synthase (DGDGS)), and (5) genes involved in plastid isoprenoid biosynthesis. We also noticed an absence of a plastid protein import or division machinery (e.g., SELMA also absent in dinoflagellates [28], MinD/ MinE proteins); as well as an absence of genes involved in the organization and expression of the plastidial genome (e.g., plastid-targeted amino-acyl tRNA synthetases) (Table S3). The availability of complete genomes for diverse organisms ranging from those harboring fully functional chloroplasts (dinoflagellates and chromerids) to those exhibiting complete loss of their plastids (*Amoebophrya*, *Cryptosporidium*) allowed us to explore the metabolic functions that were retained together with these organelles (Fig. 1). From a list of 120 metabolic pathways (Table S10, Fig. 1), we detected a few functions, linked to amino acid metabolism (ornithine and urea cycle, synthesis of isoleucine, valine and leucine, synthesis of histidine and lysine degradation) and steroid metabolism (synthesis of the squalene 2,3-epoxide), which occur only when functional chloroplasts are retained. Similarly, the isoprenoid MEP/DOXP pathway, the ferredoxin-NADP(+) reductase, the Fe-S proteins, and the oxidative phase of the pentose phosphate cycle are generally maintained when plastids persist, while the FASII pathway and the plastidial pyruvate dehydrogenase (PDH) complex, known to have key functions in *P. falciparum* and *T. gondii*, have not been retained in *Perkinsus* and piroplasmids. The maintenance of metabolic pathways for the production of several cofactors may be linked to plastid retention (vitamins B1 and B9, molybdopterin, lipoic acid), as well as the pathways for methionine salvage and the synthesis of the phenylalanine and tyrosine, which persist in *Perkinsus* but were lost in aplastidial lineages.

### Aerobic mitochondrion

Despite intensive searches in the whole-genome assemblies and transcriptomes covering a complete infection cycle for both *Amoebophrya* strains, we were unable to identify two (*cox3* and *cob*) of the canonical mitochondrial-encoded genes. However, we have identified partial candidate sequences for *cox1* similar to fragments reported from the recently published AT5 genome [23] and corresponding to the metal-binding sites located near the C-end of the protein (data not shown). These two fragments have signal peptides (according to TargetP v.2) in both *Amoebophrya* strains, with GC content (53.75–54.56% and 58.39–58.48% for A25 and A120, respectively) similar to *cox2* which is located in the nuclear genome. We recovered key components of the mitochondrial DNA replication machinery, including a homolog of plant organellar DNA polymerases (POPs). We also identified important components of the mitochondrial gene expression machinery, including a DNA-directed RNA polymerase (RPOT or RNAP), along with 31 mitochondrial ribosomal proteins (21 large and nine small subunit proteins, respectively) and a monomeric phenylalanine-tRNA (FARS2) ligase (Table S3, Fig. 1). These organellar genes were moderately to highly expressed in both *Amoebophrya* strains.

We explored whether the *Amoebophrya* mitochondrion could fulfill aerobic functions related to cellular respiration. Complex I (NADH: ubiquinone oxidoreductase) of the electron transport chain (ETC) has been replaced by an alternative non-electric NAD(P)H:ubiquinone reductase (NDH2 or NDA), and complex II succinate:ubiquinone dehydrogenase (SDH) appears to lack the two membrane-anchoring subunits SDHC and SDHD, a feature that likely evolved early in myzozoans (Fig. 1). Electron donors to the ubiquinone pool include the SDH and the electron transfer flavoprotein:ubiquinone oxidoreductase (ETFQO) complexes, the dihydroorotate: ubiquinone oxidoreductase (DHODH) protein, the glycerol 3-phosphate dehydrogenase (G3PDH) protein, NDH2, and a malate:quinone dehydrogenase (MQO). Interestingly, we found no trace of the anaerobic-related sulfide:ubiquinone oxidoreductase (SQO) in either *Amoebophrya* strains, in contrast to what has been described in chromerids. Complex III (ubiquinol:cytochrome c oxidoreductase) has also been lost, leading to a break in the ETC where the electrons from the ubiquinone pool (Q) are dissipated by an alternative oxidase (AOX) (Fig. 1). The reduction of cytochrome C is likely carried out by an L-galactono-1,4-lactone dehydrogenase (G14LDH), a membrane-bound d-lactate:cytochrome c (D-LDH), and l-lactate:cytochrome c (L-LDH or cytochrome b2) oxidoreductases. Interestingly, both dinoflagellates and closely related lineages (*Perkinsus* and *Amoebophrya*) have lost the canonical pathway to produce ubiquinone, which is still present in apicomplexans and chromerids.

Two enzymes of the OXPHOS pathway (MQO and the SDH complex) are shared with the TCA cycle in *Amoebophrya*, as described for other myzozoans [29]. The input of acetyl-CoA into the TCA cycle by conversion of pyruvate (the end-product of the glycolysis) is normally carried out by the PDH complex. The mitochondrial PDH complex was lost early in the evolution of myzozoans and replaced either by the plastidial PDH complex and/or by the branched-chain α-ketoacid dehydrogenase (BCKDH) complex [29]. The *Amoebophrya* parasites, however, lack the mitochondrial PDH, BCKDH, and the 2-oxoglutarate dehydrogenase (KGDH/OXODH) complexes, as well as canonical pathways for their two cofactors (thiamin and lipoid acid). It should be noted that a complete glyoxylate cycle in A120 (but partial in A25), as well as homologs of six core peroxins (PEX1, 5, 7, 11, 12, and 16), suggests the presence of peroxisomes in *Amoebophrya*, as it was previously described in myzozoans including Apicomplexa [30]. Other metabolic pathways usually located in peroxisomes in eukaryotes, including β-oxidation of fatty acids, catabolism of purines, and the cellular antioxidant system for the detoxification of reactive oxygen species (ROS), have also been detected in the two *Amoebophrya* strains [31].

### Non-canonical intron spreading in *Amoebophrya* genomes

**Fig. 3:**
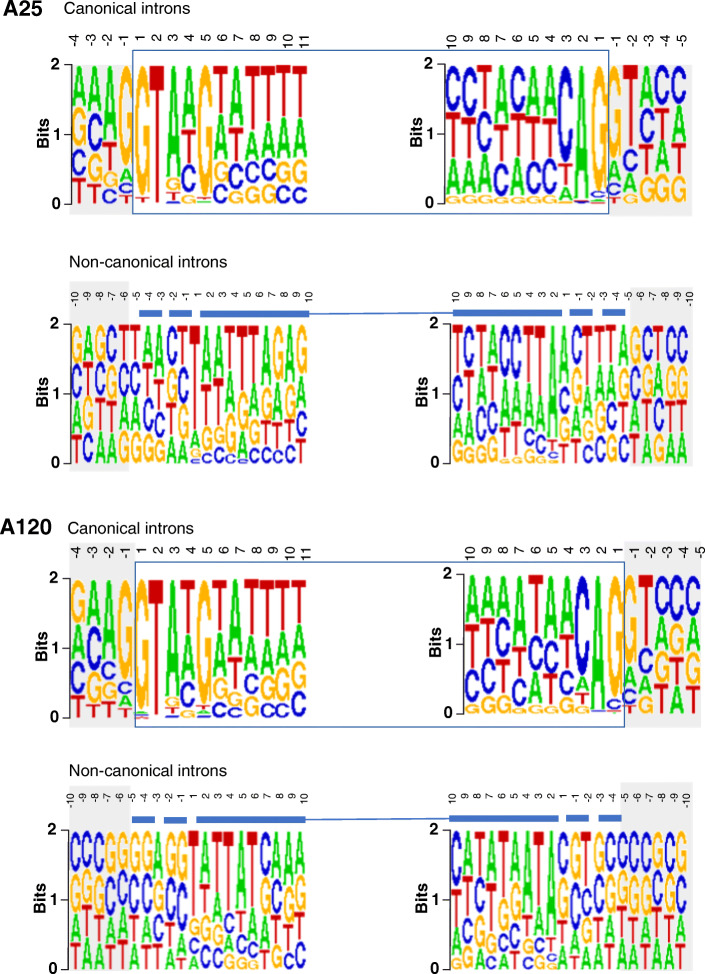
Intron splicing motifs in A25 (top panel) and A120 (bottom panel). Canonical introns: square delimiting the intron, including the canonical donor and acceptor motifs. Shaded areas up- and downstream of the intron represent exon sequence. Non-canonical introns: line above logos indicates intron region with palindromic motifs forming the hairpin (sold line). Splice sites relative to the hairpin-motif are variable (dashed line). Shaded areas represent intron border position that remains unknown

In total, we identified 55,290 and 66,565 introns supported by RNA-seq data (minimum coverage ≥ 3 reads) in the genomes of A25 and A120, respectively. Estimated intron densities (1.47 and 1.42 intron per kb of coding sequence in A25 and A120, respectively) are similar to what is commonly observed in alveolates and eukaryotes [32]. More than 60% of those in both A25 and A120 were classified as non-canonical introns (NCIs), meaning that their splice sites differed from the canonical motif GT-AG) (Table 1, Table S4). Additionally, no clear splicing signature of the two first and two last nucleotides was highlighted, indicating a low frequency for each individual combination of dinucleotide patterns at the intron-exon boundaries (Fig. 3, Table S4). Compared to canonical introns, NCIs have distinct features in terms of length and GC content (Fig. S12-S13). NCIs also differed between *Amoebophrya* strains: NCIs were smaller in A25 (120 nt on average) compared to A120 (240 nt on average, Fig. S12). We explored whether this intron prediction was affected by RNA editing [25]. Our result showed that only 2 to 4% of the total intron boundaries (within first and last 10 nucleotides of the introns, A25 and A120, respectively) might have RNA editing events (Table S5). These evidences demonstrated that if existed, these intron boundaries may not be accurately defined.

**Fig. 4:**
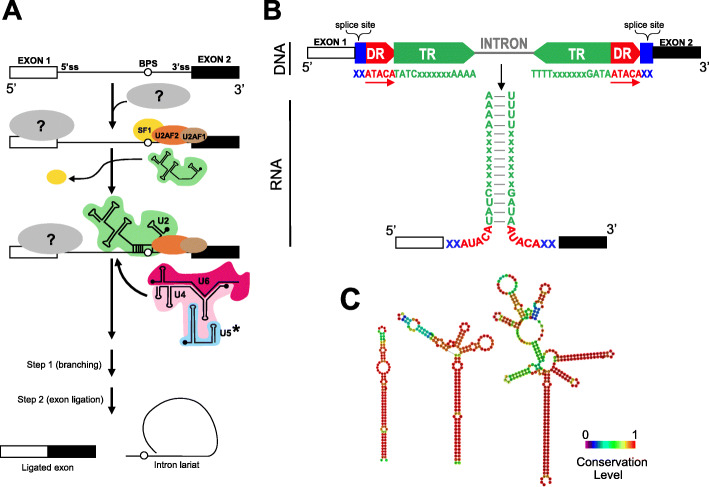
Predicted hairpin secondary structure of introners and their putative splicing mechanism. **a** Schematic representation of the splicing mechanism displaying the set of spliceosome proteins identified by sequence homology in the A25 and A120 proteomes. A missing U1 protein in both genomes is indicated by a gray area containing a question mark. * corresponds to U5 identified in A120 only. **b** A schematic structure of an introner containing direct repeat (DR) and inverted repeat (IR) motifs in the *Amoebophrya* genome (DNA). **c** Predicted secondary structure (RNA) of an introner defined by RNAfold

In both *Amoebophrya* strains, we identified nearly all protein subunits of the multimega-dalton ribonucleoprotein (RNP) complex (six out of 89 were undetectable) classically involved in the splicing mechanisms of eukaryotic introns (Table S6, Fig. 4a). The six undetected spliceosomal proteins in A25 and A120 are involved in the U4/U6 (snRNP27) and U5 (CD2BP2) complexes, in the specification of U5 and interactions with RNA (BCAS2, SYF2), and are members of the serine/arginine-rich (SR) proteins and hnRNP (heterogeneous nuclear ribonucleoprotein) families (PTBP2 and hnRNP U). Moreover, we identified all but two snRNAs, U1 (that binds the 5′-donor splice site of introns during splicing) was not detected in either A25 or A120, and U5 was missing in A25 (Fig. 4a, Figs. S14-S18). Finally, the absence of key components of the minor spliceosome (U11, U12, U4atac, and U6atac snRNAs), along with the very low proportion of introns with a canonical AT-AC splicing site, suggests the absence of this complex in *Amoebophrya* strains A25 and A120, as reported previously in other Alveolata species [33].

### Non-canonical introns (NCIs) contain a subset of introner elements (IEs)

A closer inspection revealed that about 11% (A25) and 30% (A120) of NCIs contained 8–20 nt inverted repeat (IR) motifs, forming a complementary sequence between the 5′- and the 3′-end of the same intron, and direct repeat (DR) motifs of 3–5 nt in length overlapping the exon/intron boundaries (Fig. 4b, Figs. S19-S23). We defined these repeated NCIs containing IR regions (Fig. S24) as introner elements (IEs). IR motifs can produce hairpin structures (Fig. 4b, c), allowing the joining of exon boundaries (Fig. 4b). We observed a similar organization of DR and IR motifs in 1% (A25) and 15% (A120) of canonical introns. The DRs varied in length, composition, and position: the most abundant DRs in A25 were overlapping the 5′-end and were one nucleotide downstream of the 3′-end of the IR motifs; in A120, the most abundant DRs consisted of four nucleotides upstream of the 5′-end and within two nucleotides downstream of the 3′-end of the IR motifs (Figs. S19-S23). Using hidden Markov model (HMM)-based profiles obtained from an initial set of IR motifs, we detected 2039 (20% of NCIs) and 29,850 (68% of NCIs) repeated introns representing 8 and 17% of the A25 and A120 genome assemblies, respectively. Based on IR and overall sequence similarity values, we grouped all IEs into strain-specific families (252 and 1954 families in the A25 and A120 genomes, respectively, Table S7). In A25, IR family motifs started with the conserved TTA triplet motif followed by two purines (A or G) and ended with a conserved G (Fig. S25). IR family motifs in A120 started with the TAT triplet, followed by seven less-conserved nucleotides, and ending with a minimum stretch of three conserved As (Fig. S25). We found no relationship between the remaining IR and DR-containing NCIs (28,467 and 24,976 in A25 and A120, respectively) that we classified as singletons IEs. Interestingly, we identified several identical pairs of IEs in each *Amoebophrya* genome (64 in A25; 97 in A120).

### Intron dynamics in *Amoebophrya*

We distinguished three types of genes based upon their introns: (1) genes having canonical introns only, (2) genes having NCIs only, and (3) genes having both intron types (called heterogeneous genes hereafter). Even though NCI features differed in the two *Amoebophrya* strains, the distribution of these three gene types within each strain was similar (Fig. S26). We also found the same proportion of heterogeneous genes and NCI-only genes in both *Amoebophrya* genomes (Fig. S26), suggesting a similar spreading mechanism of NCIs in A25 and A120. Moreover, the proportion of NCI-only genes with a functional annotation was similar to that for all genes (37 and 44% in A25 and A120). This value exceeded 65% for genes having canonical introns only and was similar to what is generally observed in public sequence databases (KEGG and InterPro) for heterogeneous genes. Interestingly, we found a significantly smaller proportion of IEs in genes involved in core and essential translation and ribosomal functions compared to other functional categories (Fig. S27). These observations strongly suggest a lower sequence similarity between genes having a large proportion of NCIs and known genes stored in public databases. This highlights a possible link between the presence of NCIs in genes and the evolution of their gene sequences.

When comparing intron position between orthologous genes in A25 and A120 strains, we found that 98.6% of those introns displayed canonical splice sites at conserved positions (corresponding to 19.9% and 19.4% of total introns, respectively). We observed a positive correlation between the increased portion of conserved introns and the level of protein similarity between orthologous protein gene pairs (Fig. S28), suggesting that NCIs appeared concomitantly in the respective genomes after the speciation process. By comparison, only 32.6% (A25) and 24.8% (A120) of strain-specific intron positions (found in one strain but not in the other) displayed the canonical splice site, while 20.3% and 68.5% of NCIs corresponded to IEs in A25 and A120, respectively.

## Discussion

### The *Amoebophrya* genomes are unique even among dinoflagellates

The genome sizes of the two *Amoebophrya* strains sequenced in this study (A25 and A120) were reminiscent of other parasites basal to dinoflagellates such as *Perkinsus marinus*, but ten times lower than the smallest phototrophic dinoflagellate genomes recorded to date (1.19 Gb for *Cladocopium goreaui* and 1.07 Gb for *Fugacium kawagutii*) [34]. Gene duplication is a possible explanation for this gene inflation in dinoflagellates given that the *Amoebophrya* homologous genes clustered into fewer gene families (25% and 28% in A25 and A120 respectively) than those predicted for Symbiodiniaceae (55–65%, Table 1). Moreover, the cumulative effects of a small number of transposable elements, along with short introns and intergenic regions, as well as the limited number of gene families together contribute to the compactness of the *Amoebophrya* genomes (232–273 genes/Mb) compared to other dinoflagellates (39–69 genes/Mb for Symbiodiniaceae; Table 1). Despite such differences in genome size and organization, A25 and A120 have more genes in common with Symbiodiniaceae (1945 and 1983 genes in A25 and A120 respectively) than with *P. marinus* (254 and 232 genes in A25 and A120 respectively), which adds additional evidence supporting the taxonomical classification of Syndiniales as true dinoflagellates (Fig. 1; Fig. S1).

The large proportion of species-specific genes, along with the degree of divergence in sequences predicted for the *Amoebophrya* genomes, together suggest adaptation resulting from novelty (gain of genes) rather than by reduction (loss of genes), as previously observed for other parasite models [35]. The relatively high level of SSU rDNA sequence similarity observed between the three *Amoebophrya* strains (Fig. S2) contrasts starkly with the remarkably low level of protein sequence similarity. Recent speciation between A25 and A120 must have been driven by evolutionary processes that accumulated protein sequence modifications while maintaining synteny conservation. Such a process suggests the presence of evolutionary constraints for the maintenance of gene order through a low rate of chromosomal duplication and rearrangement within the *Amoebophrya* clade, concomitant with an elevated rate of protein evolution.

The presence of a trans-spliced DinoSL motif [12] found in mature transcripts of *Amoebophrya* spp. is unique to dinoflagellates. Trans-splicing has been linked to the resolution of operons (clusters of tandemly arranged genes transcribed from a single upstream promoter into polycistronic pre-mRNAs) in kinetoplastid genomes [35] and in mRNA stability in several lineages [36]. Within an operon, all genes are constitutively transcribed into a polycistronic mRNA, where differential gene regulation happens post-transcriptionally. There is a growing consensus on post-transcriptional control of gene expression in dinoflagellates [37], while there is no evidence for polycistronic mRNAs [38] of unidirectional clusters of genes in this lineage [9]. While *Amoebophrya* genomes display a higher tendency for genes encoded by unidirectional clusters compared to Dinophyceae and Euglenozoa [9, 13, 39], no correlation between gene organization, gene function, and their expression profiles was observed during the different *Amoebophrya* developmental stages [31]. In fact, genes included within the same block displayed different expression profiles reminiscent of a pre-transcriptional regulation, with no evidence suggesting polycistronic gene co-regulation. In many organisms, DNA replication is temporally separated from transcription. This is achieved in Dinophyceae by reducing the time-frame of DNA replication, with the chromosomes remaining in a condensed state during most of interphase. This is not the case in *Amoebophrya* spp. in which sporogenesis (involving active DNA replication) starts early and occurs during most of the parasitic intracellular stage [16], in parallel with gene expression [31].

### Organelle reduction in *Amoebophrya*

*Amoebophrya* spp. have unusual organelles, where the plastid is missing and the mitogenome is either highly reduced or has been lost altogether. This is surprising given that the ancestral myzozoan obtained its plastid through tertiary endosymbiosis [3, 4], and total loss of this organelle is a rare event only observed in *Cryptosporidium* and Syndiniales [40, 41]. While several non-photosynthetic lineages still retain cryptic plastids (most apicomplexans, members of the genus *Perkinsus*, most if not all heterotrophic dinoflagellates), our results confirm the hypothesis of plastid loss early in the evolution of Syndiniales. The mitochondrial (mt) genome of dinoflagellates, apicomplexans, and relatives is drastically reduced and contains only two (cox1 and cox3 in *Chromera velia*) to three protein-coding genes (cox1, cox3 and cob in other organisms), as well as fragments of ribosomal RNA (rns and rnl) genes [41–43]. In dinoflagellates, trans-splicing of messenger RNAs (mRNAs) is required to generate complete cox3 transcripts, and extensive RNA editing recodes most genes [44, 45]. Zhang et al. [46] showed extensive frameshifts in the cox1 gene of the pathogenic alveolate *P. marinus*, which makes the identification of mitochondrial genomes very challenging in that clade. The absence of cob, as well as of the nuclear-encoded subunits of complex III (cytochrome C reductase), supports the complete loss of this complex in *Amoebophrya* (see below), a situation similar to what has been described for *C. velia* [23, 43]. A recent study reported the absence of a mitogenome in the *Amoebophrya* sp. AT5 strain, with two fragments of a cox1-like gene encoded by the nucleus, suggesting a total loss of the mtDNA in that clade [23]. The expression patterns of these cox1-like “genes” in both A25 and A120 along with the presence of mitochondrial signal peptides support the transfer of these cox1 fragments to the nucleus in *Amoebophrya*. However, split and transfer of the C-terminal domain of cox1 has been described in the amoeboid protist *Acanthamoeba castellanii* and appears to be widespread in eukaryotes [47]. Moreover, the persistence of key components of the mtDNA replication and expression machineries along with their observed expression levels are intriguing in the supposed absence of a mitogenome as suggested for AT5 [23] and suggest the likely presence of a cryptic mitochondrial genome in the two *Amoebophrya* strains A25 and A120.

We identified a complete, although highly derived, respiratory chain in both *Amoebophrya* strains similar to what was described for *C. velia* [43], with a few notable exceptions (Fig. 1). Both *Amoebophrya* strains have most enzymes for the TCA cycle, with the notable exception of all dehydrogenase complexes and the canonical pathways for their cofactors. In this context, the TCA cycle in *Amoebophrya* requires the involvement of non-canonical pathways to be functional. Anaplerotic reactions replenishing TCA cycle intermediates are possible from pyruvate via homologs of pyruvate carboxylase and malate dehydrogenase, and from phosphoenolpyruvate (PEP) via homologs of PEP carboxykinase. For instance, *Amoebophrya* is able to use glutamine (the dominant amino acid in dinoflagellates [48]) to produce oxoglutarate and fuel the TCA cycle as observed in dinoflagellates and *P. falciparum*. Moreover, the presence of a partial oxoglutarate bypass pathway (presence of the succinate-semialdehyde dehydrogenase (NAD+) [EC 1.2.1.79]) and an almost complete GABA shunt (glutamate decarboxylase is missing) in both strains that would allow the conversion of oxoglutarate to succinate is a potential way to short-circuit the missing OXODH complex.

### Singular intronic elements in *Amoebophrya* genomes

While most introns in AT5 (99.98%) were predicted to be canonical (i.e., with GT-AG splice sites [23]), more than 60% of those in both A25 and A120 were classified as non-canonical introns (NCIs), displaying a wider range of slicing site (Fig. 3). NCIs were previously observed in several eukaryotes and a deeper investigation of available genomes will help in improving our capacity to predict genes and understand splicing mechanisms [49, 50]. For instance, a recent study reported between 1.2 and 2.1% NCIs in the animal, fungal, and plant intronomes, with the motif GC-AG being the most frequent splicing site reported, followed by AT-AC (spliced by the atac spliceosome), and GA-AG. Such diversity demonstrates some flexibility at the 3′ intron splice site, with different specificities observed in each kingdom [51]. Higher proportions of NCIs were also reported in non-model organisms, such as in the tunicate *Oikopleura*, the green microalga *Micromonas pusilla*, the stramenopiles *Aureococcus anophagefferens*, euglenoids, and at least three appendicularian fritillarids [52–55]. However, all these NCIs still shared many similarities, including individual specific splicing sites. For instance, in *Fritillaria borealis* where the smallest proportion of canonical introns has been reported to date, a majority of NCIs displayed the AG-A(N) patterns. Moreover, NCIs in the two *Amoebophrya* genomes appear to favor less-conserved genes, where a larger proportion of genes with canonical introns had functional annotation and were clustered into orthologous pairs. Such distribution highlights a possible link between the presence of NCIs and the evolution of gene sequences in the two genomes.

We identified a proportion of NCIs as strain-specific introner elements (IEs) with pervasive inverted and direct repeats (IR and DR, respectively) and putative stem-loop secondary structures. Recent studies have stressed the presence of repetitive elements within introns in many organisms [53, 56, 57]. Introners have been described in the genome of the green microalgae *M. pusilla* and the stramenopile *A. anophagefferens* [53, 58], the latter IEs always displaying direct repeats (DRs) and terminal inverted repeats (TIR) of constant length and canonical splicing motifs. However, the structural peculiarities of *Amoebophrya*’s IEs, such as the extent and diversity of repeated motifs, far outpace unconventional intron splice sites [13] and identically repeated intron boundary sequences described in dinoflagellates [59].

The presence of IR and DR sequences, along with the absence of internal transposase-encoded genes, is reminiscent of non-autonomous TIR DNA transposons, where the TIR represents a unique hallmark for each DNA transposon family. DNA transposons can degenerate into non-autonomous transposable elements (commonly known as miniature inverted repeat transposable elements or MITEs) that often display short (10–15 bp) DRs resulting from target site duplications (or TSDs), and IRs, but lack transposase genes. Instead, MITEs rely on the activity of transposases encoded by cognate full-length autonomous transposons through a cut-and-paste transposition mechanism by recognizing the IR motifs for mobilization. MITEs have been detected in numerous eukaryotes including some plants, fungi, protozoans, metazoans [60, 61], and in viruses [62]. However, the presence of two putative transposases found only in A25, and not in A120, rules out the general transposase-mediated mobilization of introners in *Amoebophrya*. In addition, we found that only a small proportion of *Amoebophrya* introners (10% and 31% for A25 and A120, respectively) could be assigned to putative and yet unknown MITE families, and no family-specific IR motifs could be detected. The proportion of NCIs and the variability of the splicing sites observed within the two *Amoebophrya* genomes have thus no precedent in eukaryotes and raise the question of their splicing mechanisms. Small nuclear RNAs (snRNAs) are highly conserved components of the spliceosome in eukaryotes. For instance, the snRNA U1 subunit is involved in 5′-donor intron site recognition. The apparent loss of U1 in both *Amoebophrya* genomes suggests an alternate splicing mechanism capable of recognizing and processing unusual intron-exon boundaries, possibly through the recruitment of a novel and highly divergent protein-based subunit. Finding most snRNAs in transcriptomic data trigger the additional question of a polyadenylation of *Amoebophrya* snRNAs as found for example in *Dictyostelium discoideum* [63]. Conserved introns seem to precede a mechanism of gain or loss of NCIs, even though we cannot distinguish a gain event creating a novel intron from the loss of an ancestral intron in one of the two orthologs. Considering that 30% of NCIs are IEs in A120, it is more likely that novel introns emerged from transposon insertions (copy-paste mechanism) than by intron transposition (cut-paste mechanism) [64]. While the origin of IEs in *Amoebophrya* cannot be determined, our results suggest that the proliferation of IEs is strain-specific and still ongoing in a way arguably similar to transposable elements. Recent studies show that repetitive elements within introns are found in many organisms. Given the disparity of the IE consensus sequences between A25 and A120, IE insertion likely followed the speciation event. Yet, we predict that both *Amoebophrya* strains use the same mechanism of IE insertion, independently creating new gene structures suitable to their own species.

## Conclusions

We report here two novel genomes of *Amoebophrya* spp. (A25 and A120) parasites, the sister lineage of Dinophyceae. While these two strains are phylogenetically too distant to provide meaningful insights into parasitism and host specificity [21], they are key to understanding myzozoan evolution. Both strains share many similarities with other dinoflagellates at the genome level: their chromosomes appear to be condensed most of the time (despite the absence of a typical dinocaryon) and remain attached to the nuclear membrane [16]; they code for DVNPs [7]; some of their mature transcripts contain a truncated DinoSL motif found in other dinoflagellates [12] resulting from trans-splicing of pre-mRNAs; they share more orthologous genes with Dinophyceae than with any other myzozoan. However, *Amoebophrya* differ from Dinophyceae by several genomic features, the most prominent ones being the compactness of their genomes, the loss of their plastid, and the reduction of their mitochondrion. For instance, the concomitant loss of all dehydrogenase complexes has no precedent in myzozoans retaining a functional TCA. This essential metabolic pathway may still persists thanks to the retention of alternative pathways also detected in sister lineages and likely inherited from a myzozoan ancestor. The cumulative effect of a small number of transposable elements, along with short introns and intergenic regions, and the limited number of gene families all contribute to the compactness of the *Amoebophrya* genomes when compared to other dinoflagellates. A compact genome and the strong synteny observed between the two strains suggest a long-term evolutionary constraint on chromosome organization within the *Amoebophrya* clade in contrast to what was observed in Symbiodiniaceae. Meanwhile, the low values of protein sequence similarity are potentially linked to parasitism, as this way of life often coincides with relaxed functional constraints leading to higher substitution rates [65]. The non-canonical splicing sites, the large diversity of size, and DR motifs make the *Amoebophrya* introners (IEs) a novel type of repetitive element for which the splicing mechanism should be distinct from the ubiquitous eukaryotic splicing machinery. *Amoebophrya* IEs can form putative stem-loop secondary structures that may be involved in their mobilization. Such mechanisms common to both *Amoebophrya* strains must have preceded their divergence, enabling the retention and proliferation of IEs. Taken together, our results suggest that the sequencing of additional *Amoebophrya* genomes and transcriptomes is required for the exploration of the origin and spread of NCIs and IEs, and also to investigate their potential impact on protein evolution. Overall, additional well-annotated genomes from other basal Syndiniales will shed light on the mechanisms underlying the atypical and contrasting genome organizations observed in dinoflagellates, i.e., from constrained highly compact genomes to relaxed gigantism.

## Methods

### Origin of strains and stock culture

We obtained all strains from the Penzé estuary (North-West of France, English Channel, 48° 37′ N; 3° 56′ W) and cultivated them using F/2 medium (Marine Water Enrichment Solution, Sigma), prepared with filtered and autoclaved natural seawater from the Penzé estuary, and complemented with 5% (v/v) local soil extract. We maintained all stock cultures at 19 °C and on an L:D cycle of 12:12 h at 80 μEinstein m^2^ s^− 1^. A protocol detailing A25 and A120 cell harvesting for genomic and transcriptomic analyses can be found at the protocole.io dx.doi.org/10.17504/protocols.io.vrye57w.

### Short-read Illumina library preparation and sequencing

DNA was quantified on a Qubit Fluorometer using the Quant-iT dsDNA Assay Kit (Life Technologies, Carlsbad, California, USA), and its quality was checked by electrophoresis in a 0.7% agarose gel. For both strains, an overlapping paired-end (PE) library and a mate-pair library (MP) were prepared for Illumina sequencing. PE overlapping library preparations were carried out from 250 ng of genomic DNA using a semi-automated protocol. Briefly, DNA was sheared with the Covaris E210 instrument (Covaris, Inc., Woburn, Massachusetts, USA) to generate fragments of 150–400 bp. End repair, A-tailing, and ligation with Illumina compatible adaptors (Bioo Scientific Austin, Texas, USA) were performed using the SPRIWorks Library Preparation System and a SPRI-TE instrument (Beckmann Coulter, Danvers, Massachusetts, USA) according to the manufacturer’s protocol. Fragments of 200–400 bp were selected and amplified by 12 cycles of PCR with the Pfx Platinum Taq polymerase (Thermo Fisher, Waltham, Massachusetts, USA) and Illumina adapter-specific primers. Amplified library fragments of about 300 bp were selected (second round of selection) on 3% agarose gel and purified.

For strain A25, a mate-pair (MP) library was prepared according to the initial Illumina protocol (Illumina Mate Pair library kit, Illumina, San Diego, CA) with approximately 10 μg of genomic DNA subjected to Covaris fragmentation. For strain A120, the MP library was prepared with the Nextera Mate Pair Sample Preparation Kit (Illumina) using 4 μg genomic DNA that was simultaneously fragmented by enzymatic treatment and tagged with a biotinylated adaptor. The resulting fragmented and tagged (tagmented) DNA was subjected to size selection (8–11 kb) by gel electrophoresis and circularized by overnight incubation with a ligase. Linear, non-circularized fragments were digested, while circularized DNA was fragmented to generate fragments of 300–1000 bp with the Covaris E210 system. Biotinylated DNA was immobilized on streptavidin beads, end-repaired, 3′-end adenylated, and ligated with Illumina adapters. DNA fragments were amplified by PCR with Illumina adapter-specific primers and purified. The quality of all Illumina libraries was evaluated with an Agilent 2100 Bioanalyzer (Agilent Technologies, Palo Alto, CA, USA) and quantified by qPCR with the KAPA Library Quantification Kit (KapaBiosystems Inc., Woburn, MA, USA) on a MxPro instrument (Agilent Technologies). Libraries were sequenced using 101-bp PE reads chemistry on a HiSeq2000 Illumina sequencer. All Illumina PE and MP reads were cleaned through a four-step process using fastx_clean (http://www.genoscope.cns.fr/fastxtend), an in-house software based on the FASTX toolkit (http://hannonlab.cshl.edu/fastx_toolkit/), by discarding (i) sequencing adapters and low-quality nucleotides (quality value < 20); (ii) sequences located between the second unknown nucleotide (N) and the end of the read; (iii) reads shorter than 30 nucleotides after trimming; (iv) reads and their mates mapping onto run quality control sequences (the PhiX genome).

### Long-read Nanopore library preparation and sequencing

Genomic DNA was size selected (10–50 kb for both organisms and 20–80 kb cut-offs for A120 only) using a BluePippin (Sage Science, Beverly, MA, USA) and repaired depending upon the DNA quantity recovered using the NEBNext FFPE Repair Mix (New England Biolabs, Ipswich, MA, USA). Following end-repair and 3′-A-tailing with the NEBNext® Ultra™ II End Repair/dA-Tailing Module (NEB), sequencing adapters provided by ONT (ONT Ltd., UK) were ligated using Blunt/TA Ligase Master Mix (NEB). Each library was then mixed with the running buffer with “fuel mix” and the library loading bead, and loaded on MinION R9.4 SpotON Flow Cells. Two and three libraries were run for the A25 and A120 strains, respectively. Read event data were generated by the MinKNOW control software (successive versions 1.3.25, 1.3.30, then 1.4.3 have been used) and base-calling done with the Metrichor software version 2.43.1, then 2.45.3 (1D base-calling RNN for LSK108 workflow). The data generated (pores metrics, sequencing, and base-calling data) by the MinION software was stored and organized using a Hierarchical Data Format. FASTA reads were extracted from MinION Hierarchical Data Format files using poretools [66].

### Genome size estimation

We estimated the genome sizes of the two parasitic strains using both flow cytometry and k-mer analysis. For flow cytometry, nuclei were extracted by mixing 50 μL of freshly produced dinospore with 450 μL of 0.25X NIB buffer [67], containing SYBR Green-I at a final concentration of 1/5000. We used 2 μL of a culture of exponential growing *Micromonas pusilla* RCC299 (1C = 20.9 fg) as an internal reference. The mixture was then incubated for at least 30 min in the dark before being analyzed using a FACS Canto II flow cytometer equipped with a 488-nm laser and the standard filter setup, where the signal was triggered by green fluorescence. The ratio between the mean distribution of the dinospores and the RCC299 was used for the evaluation of the DNA content. K-mer size estimation was calculated considering Illumina 100 bp paired-end reads using Jellyfish [68] with the following parameters: -m 31 -s 2048M –C to generate a 31-mer distribution and the K-mer histogram was uploaded to the GenomeScope website (http://qb.cshl.edu/genomescope/).

### Genome assembly

We used both short Illumina and long Nanopore reads to generate genome assemblies for the two *Amoebophrya* strains. First, we obtained a draft Illumina-based assembly from the combination of Illumina paired-end and mate-pair reads using the All-PathsLG [69] program with default parameters. Gaps were closed using GapCloser from the SOAPdenovo package [70]. In order to detect and remove chimeric junctions that are present in Illumina scaffolds, we aligned Nanopore reads on the Illumina assemblies using the Last aligner package [71]. Then, we used NanoSV [72] to detect any mis-mapping in reads that could indicate a chimeric scaffold. Finally, we cut the scaffold sequences at each breakpoint indicated by NanoSV. Second, we generated a Nanopore-only draft assembly for each genome. For A25, we used all Nanopore reads (corresponding to an estimated 23× genome coverage) as inputs to the SMARTdenovo assembler (Jue Ruan, Ultra-fast de novo assembler using long noisy reads, 2016, available at https://github.com/ruanjue/smartdenovo) with the –k 17 to increase k-mer size (as advised by the developers on large genome sizes) and –c 1 to generate a consensus parameters. For A120, we selected the longest Nanopore reads corresponding to an estimated 30× (out of 120×) coverage of the genome as input to the SMARTdenovo assembler as previously described [26, 73] with the –k 17 and –c 1 parameters. Then, we aligned the Illumina short reads onto the Nanopore assemblies using BWA mem [74] in order to correct non-random mainly homopolymeric Nanopore errors, and gave the resulting alignments as input to Pilon [75] in order to correct the consensus of the Nanopore-only assemblies. Finally, we decided to preserve the original Illumina scaffolds generated by ALLPATHS-LG assembler by organizing them into super-scaffolds based on the Nanopore-only assemblies. We aligned the Illumina scaffolds of each genome onto its respective Nanopore-only assembly using Nucmer [76] and kept only the best match with the delta-filter command. We considered a match only if the alignment covered more than 90% of the Illumina scaffold with at least 85% identity. Thanks to this list of matches, we organized the Illumina scaffolds along the Nanopore assemblies as the final assembly for gene annotation.

### Transcriptome assembly

We filtered the raw transcriptome data from a previous study [31] in order to remove clusters composed by transcripts that are highly expressed, and ribosomal RNA-like reads were excluded using the SortMeRNA program [77]. All reads from each time point were pooled before producing transcriptome assemblies for several life stages of each parasite using oases v. 0.2.08 [78] with a k-mer size of 51. We cleaned the assemblies with dustmasker from the ncbi-blast-2.2.27+ toolkit [79] and trimmed the 5′ and 3′ low-complexity ends. RNA-seq reads were aligned against the assembled transcripts (Table S8), and the assembled transcripts were aligned against the genome assembly (Table S9) (each organism transcript sequence has been mapped against their corresponding genome). Contigs longer than 150 bp and containing more than 75% of unmasked nucleotides from all transcriptomes were kept and used for the gene prediction of each genome separately.

### Gene prediction

A first attempt to align the assembled transcriptomes against the *Amoebophrya* genomes revealed an unusually high rate of non-canonical splice sites, rendering the use of classical mappers and ab initio gene prediction software unfit for annotating the *Amoebophrya* genomes. We therefore developed an in-house annotation pipeline based on transcriptomes to take into account the non-canonical introns whose splice sites were confirmed by the RNA-seq data. Most of the genome comparison analyses described below were performed on repeat-masked sequences using the following tools: RepeatMasker version 3.3.0 [80] to look for known repeats and transposable elements from alveolates included in the RepBase database [81]; TRF version 4 [82] for the tandem repeats; DUST [79] for low-complexity repeats. In parallel, we also performed ab initio detection of repeat patterns with RepeatScout [83].

In a first approach, the transcriptomes obtained for the life stages of the parasites were mapped onto the respective genome assemblies using the program EST2GENOME [84]. But, given that EST2GENOME expects canonical GT-AG splicing sites, we explored the possibility of alternative exon-intron boundaries by aligning the transcripts to the genome assemblies with BLAT (≥ 90% sequence identity and ≥ 85% aligned query length), keeping only the best match per transcript. Moreover, 456,355 alveolate proteins downloaded from the UniProtKB [85] databank (9/2014) were aligned to the genome assemblies using BLAT [86]. Subsequently, we extracted the genomic regions without protein hits and realigned the Uniprot proteins with more permissive parameters using BLAST [87]. Each significant match was then refined using Genewise [88] in order to refine exon/intron boundaries. Given that Genewise settings use a canonical splice site model, these protein alignments were essentially used to find open reading frames (ORFs). Alignments of *Amoebophrya* assembled transcripts and conserved proteins were used as input to Gmove [89], an in-house combiner program, to predict gene models for both A25 and A120 strains. Briefly, putative exon and intron boundaries extracted from the alignments were used to build a simplified graph by removing redundancies. Then, Gmove extracted all paths from the graph and searched ORFs consistent with the protein alignment evidence. Finally, a selection step was made for all candidate genes based on gene structure, where the model with the longest (> 100 nt) ORF per coding locus was selected. Intron-less genes (with ORF < 300 nt in size), as well as overlapping spliced genes, were removed. Completeness of the predicted gene was done using the Eukaryote set of the BUSCO database (version 4.0.2, Eukaryotic dataset, [27]) and by remapping RNA-seq reads.

### Functional annotation

Domains were defined using InterProScan [90] for both *Amoebophrya* proteomes. Moreover, we assigned functional categories to these *Amoebophrya* proteomes using the Biomolecular Relations in Information Transmission and Expression (BRITE) functional hierarchies from the KEGG database [91] as described elsewhere [31]. In order to ensure the reproducibility of our annotation approach, we re-annotated the proteomes of the coral symbiont *Fugacium kawagutti*, the malaria parasite *Plasmodium falciparum* and the perkinsozoan *Perkinsus marinus* using the same strategy. We then scored the completeness of KEGG pathways in each organism by estimating the fraction of predicted enzymatic reactions present in the query organism when compared to the canonical pathways defined by the KEGG database using the KEGG MODULE reconstruction pipeline with default parameters [91]. We checked missing annotations of the major metabolic pathways in our genomes by comparing them to those of *Toxoplasma gondii* obtained from the (Liverpool) Library of Apicomplexan Metabolic Pathways (LAMP; http://www.llamp.net/), and of *P. falciparum* obtained from the Parasite Metabolic Pathways (MPMP; http://mpmp.huji.ac.il/). We validated the identity of candidate genes by the presence of functional domains and sequence alignments with closely related proteins.

### Building gene families

Gene family analyses were conducted by comparing the predicted proteomes of both *Amoebophrya* A25 and A120 strains with those of twelve other protist species: the symbiotic dinoflagellates *Fugacium kawagutii* ( [92]; http://web.malab.cn/symka_new/), *Breviolum minutum* ( [13]; http://marinegenomics.oist.jp/symb/viewer/info?project_id=21), and *Symbiodinium microadiaticum* ( [93]; http://smic.reefgenomics.org/); the perkinsids *Perkinsus marinus* (http://protists.ensembl.org/Perkinsus_marinus_atcc_50983/Info/Index); the apicomplexans *Plasmodium falciparum* strain 3D7 ( [94]; http://plasmodb.org/plasmo/), *Toxoplasma gondii* strain ME49 ( [95]; http://toxodb.org/toxo/), *Chromera velia* strain CCMP 2878 ( [96]; http://eupathdb.org/), *Vitrella brassicaformis* strain CCMP 3155 ( [96]; http://eupathdb.org/), *Theileria equi* ( [97]; http://eupathdb.org/), and *Cryptosporidium parvum* ( [98]; http://cryptodb.org/cryptodb/); the kinetoplasts *Trypanosoma brucei* strain TREU 927 [99]; http://tritrypdb.org/tritrypdb/ release 9.0) and *Leishmania major* strain Friedlin; http://tritrypdb.org/tritrypdb/). We performed all-against-all BLASTp searches (*E* value = 1e−5; min. alignment length of the shortest protein = 50%) for all fourteen proteomes using the NCBI Blast+ 2.2.28 package, and clustered the proteins into OrthoGroups (OG) using a Markov cluster (MCL 14-137) algorithm [100].

### Define syntenic clusters

Pairwise protein alignment was done using the Smith-Waterman algorithm (https://kundoc.com/pdf-automatic-analysis-of-large-scale-pairwise-alignments-of-protein-sequences-.html) (BLOSUM62, gapo = 10, gape = 1) for all alveolate species (the three *Amoebophrya* strains A25, A120, and AT5, three Symbiodiniaceae species (*F. kawagutii*, *S. microadiaticum,* and *B. minutum*)*, P. marinus*, and *P. falciparum*), retaining alignments with a score > 300. From these alignments, orthologous and paralogous genes were identified using a Best Reciprocal Hits (BRH) approach. In order to evaluate the degree of the selective pressure of a protein-coding gene between both *Amoebophrya*, we calculated the dN/dS ratio using KaKs_Calculator1.2 with the MA (model average) method. On another hand, orthologs between two species were clusterized depending on their localization on their respective genomes. Then, each cluster, corresponding to a syntenic region, was defined as containing at least five consecutive genes and allowing a maximum distance of fifteen genes between any two genes. All syntenies were represented as a dot-plot graph where a dot is an ortholog gene pair.

### Detecting tandem duplication

We inferred tandemly duplicated genes in both *Amoebophrya* A25 and A120 genomes by comparing the protein sequences of predicted genes in each genome, and homolog pairs were retained only if they shared ≥ 95% identity at the protein level with a minimum alignment length of 90% of the total longest protein length. Then, proteins were grouped according to their similarity values using a single linkage clustering algorithm. For each cluster, two genes were defined as co-localized if they were contiguous by their rank (i.e., genomic location) on the genome, where only one gene without match against the genes in the same cluster was allowed between the pair.

### Clusters of co-oriented genes

We computed the distribution of gene orientation changes for all three Symbiodiniaceae (*F. kawagutii*, *B. minutum*, and *S. microadiaticum*) and *Amoebophrya* (A25, A120, and AT5) strains using a non-overlapping 10-gene sliding window [93]. We defined co-oriented gene blocks of at least five contiguous genes (based on their rank along the genome sequences) with the same orientation and a maximum of two contiguous genes in an opposite orientation.

### Detection of trans-spliced genes

In order to identify putative trans-spliced genes in *Amoebophrya* A25 and A120 genomes, we searched the 16 nt 3′-end region of the dinoflagellate spliced leader (DinoSL) sequence in the RNA-seq data using a k-mer approach with kfir (www.genoscope.cns.fr/kfir) and a k-mer size equal to 8. The reads containing the DinoSL-like motifs were aligned against their respective genome assembly using BWA mem [74]. Only the reads containing the last 5 nt (TCAAG) of the DinoSL were later selected among the soft-clipped part of the alignments. In order to define the SL sequence for both *Amoebophrya* A25 and A120 strains, we extended up to 13 nt upstream toward the 5′-end soft-clipped position in the genome without divergence from the DinoSL consensus sequence. The first match after the soft-clipped region in the RNA-genome alignment was considered as the putative SL junction. If the two last bases before this position did not correspond to the DinoSL 3′-end “AG” dinucleotides, the putative SL junction was shifted upstream while the DinoSL sequence was manually verified. We then used a multiple sequence alignment approach in order to define the consensus SL sequence for each *Amoebophrya* A25 and A120 strain. Finally, we compared the locations of these putative SL junctions on the genome assemblies with our gene predictions. A putative SL junction was associated with a gene either if it overlapped the 5′ UTR region of the corresponding gene or the first coding exon. The putative SL junctions located in intergenic regions were linked to the nearest gene models.

### Intron analyses

We obtained RNA-seq validated intronic sequences with Hisat2 (--very-sensitive --qc-filter --max-intron length 10000 [101]) and Regtools (junctions extract -a 8 -i 40 -I 10000 [102];). Only introns validated with a minimum coverage of three RNA-seq reads at the splice junctions and a length window of 40–1000 bp were used for further analyses. We used a consensus canonical motif to differentiate canonical introns from non-canonical introns (NCIs). NCIs were compared to each other using BLASTn (all-against-all, *E* value = 1e−5 [87];) and clustered using OrthoMCL (*I* = 5, [103]). All intronic sequences from each cluster were subsequently aligned with MUSCLE (v. 3.8.31, -diags) [104]. We used the PatScan software v.20110223 [105] to identify conserved palindrome motifs (referred to as inverted repeats, IRs) around the splice sites. We then regrouped NCIs into families based on their IRs (100% identity in sequence composition and length) and intronic (identity ≥ 30%) sequences using the CD-HIT program [106]. We constructed HMM profiles for each repeated NCI (introner or IE) family using hmmbuild (*E* value = 1e−5) from the HMMER v. 3.1b package [107]. To classify the super families of introners, we used hierarchical clustering (hclust, method = euclidean, ward. D) in R (v 3.2.2). We estimated the percent identity and the length of the IEs using the “Needle” sequence aligner from the Emboss v. 6.1.0 package [108] and analyzed the median percent identity and length using the ggplot2 and ggdendro scripts from the R packages.

### Conserved introns between orthologous genes

We compared intron position conservation between orthologous genes for *Amoebophrya* A25 and A120 strains by building homologous protein gene alignments with Muscle v3.7 [104], and filtering out highly variable positions with Gblocks (v0.91b). We tagged the last amino acid of each spliced exon in the alignments and considered any intron as conserved if it was present at the same location in the two orthologous proteins, in the same phase and conserved block in the alignment.

### Transposable elements

We annotated repetitive elements in the *Amoebophrya* genomes using the REPET package [109]. We also built libraries of consensus sequences representative of repetitive elements found in the A25 and A120 assemblies separately using the TEdenovo pipeline [109], and used these libraries to annotate similar regions in the assemblies using the TEannot pipeline [110]. We searched for putative transposase genes that may mediate the movement of repetitive elements by building a library of conserved protein domains belonging to DNA transposons from the Repbase database [81]. We used this library as a query to search the A25 and A120 assemblies by reverse position-specific (RPS) BLAST searches. We also used detect MITE [111] to identify the putative MITE elements in two genomes.

### RNA editing in introns

Positions with potential RNA editing have been screened in the two genomes while minimizing false positive signals using the following steps: (1) we retained positions localized in genomic regions where both the DNA and the RNA sequenced reads have unique match during mapping and (2) by using REDItools version 2.0 using the script REDItoolDnaRnav13.py (https://github.com/BioinfoUNIBA/REDItools/blob/master/NPscripts/REDItoolDnaRnav13.py), we removed positions having DNA SNPs and retained only those having a frequency up to 40% and 45% for A25 and A120, respectively; (3) we finally removed positions included within repeated elements. Then, we counted the number of remaining positions located in introns, and estimated their proportion falling at the beginning or the end of introns.

### Spliceosome component

The orthologous genes between A25 and A120 *Amoebophrya* and *P. falciparum*, *T. gondii*, and *H. sapiens* small nuclear ribonucleoproteins (snRNPs) [112, 113] were detected using orthologs defined as BRH. All identified orthologs in A25 or A120 were kept when more than one protein was found. Moreover, the Markov cluster algorithm (MCL 14-137) [100] was used to identify other snRNPs proteins in A25 and A120 genomes. Briefly, the best match of *Amoebophrya* proteins with each reference of snRNPs from *P. falciparum* and *T. gondii*, in a same MCL cluster, was selected as a snRNP prediction. Finally, the orthologs between *Amoebophrya* A25 and A120 were used to verify and complete the detection of the snRNPs.

The U1, U2, U4, U5, and U6 snRNAs were searched in *Amoebophrya* A25 and A120 genomes. For that, a BLASTN [87] was performed on the *Amoebophrya* genomes of A25 and A120 using *P. falciparum*, *S. minutum*, *H. sapiens*, and *S. cerevisiae* snRNA sequences as queries with the default parameters. Only the U6 snRNA of these organisms was found in A120 genome at 9 different loci, whereas 7 U6 genes and one single copy of U4 were detected in *Amoebophrya* A25. The U1, U2, and U5 snRNAs were neither found in A25 nor in A120 genomes using this method. Therefore, a BLASTN of the snRNA references was performed against *Amoebophrya* A25 and A120 assembled contigs of RNA-seq of all samples. In total, 18 and 26 matches were retained (A25 and A120 respectively) after choosing the best match per transcript non-overlapping regions. For each result, a BLASTN against the RNA-seq sample (host only) was performed in order to eliminate transcripts belonging to the host. Moreover, each predicted snRNA sequences left was verified by genomic coverage of each genome reads. As a result, 12 and 18 snRNAs were predicted for both *Amoebophrya* A25 and A120 respectively. U1 snRNA was not found in each organism. U5 snRNA was found only in *Amoebophrya* A120. U2, U4, and U6 were found in both organisms with this method. Figure S12 to S15 show the multiple alignments of A25 and A120 snRNA predictions and *P. falciparum* and *H. sapiens* snRNAs using muscle algorithm with default parameters [104] and Boxshade (http://www.ch.embnet.org/software/BOX_form.html) for the visualization. Each of these snRNA sequences from A25 and A120 were validated by structural conformation with known U2 snRNA structure (in particular human U2 snRNA) using Infernal software with Rfam12 database. Figure S18 shows the secondary structure of each snRNA found in both *Amoebophrya* A25 and A120 in comparison with *H. sapiens* snRNAs using VARNA software for the visualization.

## Supplementary information

**Additional file 1: Figure S1.** Phylogeny of Alveolata. Proteomes from 89 alveolates genomes and transcriptome assemblies from the MMETSP project (https://zenodo.org/record/257026/files/) were used to create orthologous groups using orthofinder v2.2 with the diamond BLAST similarity search. Single ortholog alignments were pruned using PhyloTreePruner v.1.0 (minimum taxa to keep 44 and support value 0.9) and realigned using mafft v7 and filtered with Gblocks v.0.91b (−b5 = a -p = n). Filtered alignments were concatenated using seqCat.pl and a phylogenetic tree was produced under Maximum Likelihood framework using RAxML v8.2.9 with the PROTGAMMALGF model of sequence evolution and 101 bootstraps. Asterics represent support values of 95 and above. A detailed method can be found in Kayal et al. 2018 BMC Evol. Biol. (https://doi.org/10.1186/s12862-018-1142-0). The full tree can be found at http://mmo.sb-roscoff.fr/jbrowseAmoebophrya/. **Figure S2.** SSU rDNA sequence identity (in percentage, relative to A25 and A120 compared to other species). **Figure S3.** Distribution of k-mer in A25 and A120 genomes. **Figure S4.** Classification of repeated elements in 3 *Amoebophrya* genomes (AT5, A25, and A120) using REPET. The x-axis represents the cumulated number of bases of repeated elements in the genome. **Figure S5.** Conserved motif of the putative splice leader (SL) in A25 and A120. **Figure S6.** Alignments of gene encoding the putative *spliced leader* (SL) gene in A25 and A120. **Figure S7.** Gene orientation change rate in 3 *Amoebophrya* genomes. **Figure S8.** Number of orthologs genes shared by selected taxa. **Figure S9.** Boxplot of the dN/dS ratios of orthologous genes between A25 and A120, calculated using the model average method (MA). **Figure S10.** Synteny dot-plot obtained by comparison between *Amoebophrya* A25 and AT5 genomes. **Figure S11.** Synteny dot-plot obtained by comparison between *Amoebophrya* A120 and AT5 genomes. **Figure S12.** Intron length distribution. **Figure S13.** GC content distribution. **Figure S14.** Multiple alignments of U2 snRNAs. **Figure S15.** Multiple alignments of U4 snRNAs. **Figure S16.** Multiple alignments of U5 snRNAs. **Figure S17.** Multiple alignments of U6 snRNAs. **Figure S18.** Secondary structure of *Amoebophrya* snRNA. **Figure S19.** Example of introner elements (IEs) in *Amoebophrya*. **Figure S20.** Distribution the direct repeats with size ranging between 3 and 8 nucleotides in A25. **Figure S21.** Distribution of the direct repeats with size ranging between 3 and 8 nucleotides in A120. **Figure S22.** Composition of direct repeats in introners elements. The diversity in composition of the three (a, b, c) most abundant of direct repeats in introner elements in A25 (up) and A120 (down). **Figure S23.** Terminal inverted repeat locations around the splicing sites in A25 and A120. The position of inverted repeats according to the location of the splice sites in A25 and A120. Left, the inverted repeats of A120 are located at 1–5 the nucleotides upstream and downstream of the splice sites. Right, the inverted repeats of A25 are located at the 1–6 nucleotides in upstream and downstream of the splice sites. **Figure S24.** The flowchart for the in silico search of introner elements. **Figure S25.** Hierarchical clustering analysis (pairwise similarity and OrthoMCL) of all intron families and of the inverted repeats in A25 and A120. **Figure S26.** Percentage of genes with assigned functions in relation with introns composition. **Figure S27.** Difference in the proportion of IEs-containing-genes compared to their KEGG assignment in A25 and A120. **Figure S28.** Distribution of conserved introns. **Table S1.** RCC number, date and site of isolation of strains considered in this study. **Table S2.** Metrics of Nanopore runs for the two *Amoebophrya* strains. **Table S3.** Search for pathways involved in plastidial functions that are entirely independent of plastid-encoded gene content. **Table S4.** Number of the different types of introns identified in A25 and A120 genomes. **Table S5.** Search for RNA editing in A25 and A120 introns. **Table S6.** Putative *Amoebophrya* A25 and A120 snRNP homologs. **Table S7.** Classification into families of non-canonical introns in A25 and A120. **Table S8.** RNAseq read assembly statistics of *Amoebophrya* A25 and A120 corresponding samples from the different time of infection and to the free-living stage (dinospore only). **Table S9.** Total number of contigs belonging to samples from different stages of infection and the proportion of them that were aligned against the genomes of both *Amoebophrya* A25 and A120. ND corresponds to “not determined” when no measurement was done. **Table S10.** Metabolic pathway screened in A25 and A120 proteomes.

## Data Availability

The genome data have been submitted to EMBL (BioProject PRJEB39972) [114]. A genome browser that additionally provides structural and functional annotations is also available (http://application.sb-roscoff.fr/blast/hapar/, [115]).

## Abbreviations

DinoSL: Dinoflagellate spliced leader
MALV: Marine ALVeolate
ONT: Oxford Nanopore Technologies
KEGG: Kyoto Encyclopedia of Genes and Genomes
OG: OrthoGroups
NCI: Non-canonical intron
IR: Inverted repeat
DR: Direct repeat
IE: Introner element
PDH: Pyruvate deHydrogenase
TCA cycle: Tricarboxylic acid cycle (or the Krebs cycle)
ETC: Electron transfer chain
BCKDH: Branched-chain α-ketoacid dehydrogenase
OXODH: 2-OXOglutarate dehydrogenase
PEP: Phosphoenolpyruvate
ROS: Reactive oxygen species
OXPHOS: Oxidative phosphorylation
