# Author Correction to: Rapid protein evolution, organellar reductions, and invasive intronic elements in the marine aerobic parasite dinoflagellate *Amoebophrya* spp

**DOI:** 10.1186/s12915-021-01144-8

**Published:** 2021-09-22

**Authors:** Sarah Farhat, Phuong Le, Ehsan Kayal, Benjamin Noel, Estelle Bigeard, Erwan Corre, Florian Maumus, Isabelle Florent, Adriana Alberti, Jean-Marc Aury, Tristan Barbeyron, Ruibo Cai, Corinne Da Silva, Benjamin Istace, Karine Labadie, Dominique Marie, Jonathan Mercier, Tsinda Rukwavu, Jeremy Szymczak, Thierry Tonon, Catharina Alves-de-Souza, Pierre Rouzé, Yves Van de Peer, Patrick Wincker, Stephane Rombauts, Betina M. Porcel, Laure Guillou

**Affiliations:** 1 Génomique Métabolique, Genoscope, Institut François Jacob, CEA, CNRS, Univ. Evry, Université Paris-Saclay, 91057 Evry, France; 2 School of Marine and Atmospheric Sciences, Stony Brook University, Stony Brook, New York, 11794 USA; 3 Department of Plant Biotechnology and Bioinformatics, Ghent University, Ghent, Belgium; 4 VIB Center for Plant Systems Biology, Ghent, Belgium; 5 Sorbonne Université, CNRS, FR2424, Station Biologique de Roscoff, Place Georges Teissier, 29680 Roscoff, France; 6 Sorbonne Université, CNRS, UMR7144 Adaptation et Diversité en Milieu Marin, Ecology of Marine Plankton (ECOMAP), Station Biologique de Roscoff SBR, 29680 Roscoff, France; 7 URGI, INRA, Université Paris-Saclay, 78026 Versailles, France; 8 Unité Molécules de Communication et Adaptation des Microorganismes (MCAM, UMR7245), Muséum national d’Histoire naturelle, CNRS, CP 52, 57 rue Cuvier, 75005 Paris, France; 9 Sorbonne Université, CNRS, UMR 8227, Station Biologique de Roscoff, Place Georges Teissier, 29680 Roscoff, France; 10 Centre for Novel Agricultural Products, Department of Biology, University of York, Heslington, York, YO10 5DD UK; 11 Algal Resources Collection, MARBIONC, Center for Marine Sciences, University of North Carolina Wilmington, 5600 Marvin K. Moss Lane, Wilmington, NC 28409 USA; 12 Department of Biochemistry, Genetics and Microbiology, University of Pretoria, Pretoria, South Africa

**Author Correction to: BMC Biology 19, 1 (2021)**

**https://doi.org/10.1186/s12915-020-00927-9**

Following publication of the original article [1], the authors would like to correct the affiliations of Phuong Le, Pierre Rouzé, Stephane Rombauts and Yves Van de Peer:

3 Center for Plant Systems Biology, VIB, Ghent, Belgium, & Department of Plant Biotechnology and Bioinformatics, Ghent University, Ghent, Belgium

should be split in two affiliations:

3 Department of Plant Biotechnology and Bioinformatics, Ghent University, Ghent, Belgium

4 VIB Center for Plant Systems Biology, Ghent, Belgium

and ‘University’ of should be added to:

12 Department of Biochemistry, Genetics and Microbiology, Pretoria, South Africa

to become:

12 Department of Biochemistry, Genetics and Microbiology, University of Pretoria, Pretoria, South Africa

The affiliation list has been updated in this Correction article and the original article [1] has been corrected.
