# Supplementary material for: Rapid protein evolution, organellar reductions, and invasive intronic elements in the marine aerobic parasite dinoflagellate Amoebophrya spp
Source: BMC Biol. 2021 Jan 6;19:1. doi: 10.1186/s12915-020-00927-9 (PMC7789003; doi:10.1186/s12915-020-00927-9)
Supplement: Supplementary file 1 — Additional file 1: Figure S1. Phylogeny of Alveolata. Proteomes from 89 alveolates genomes and transcriptome assemblies from the MMETSP project (https://zenodo.org/record/257026/files/) were used to create orthologous groups using orthofinder v2.2 with the diamond BLAST similarity search. Single ortholog alignments were pruned using PhyloTreePruner v.1.0 (minimum taxa to keep 44 and support value 0.9) and realigned using mafft v7 and filtered with Gblocks v.0.91b (−b5 = a -p = n). Filtered alignments were concatenated using seqCat.pl and a phylogenetic tree was produced under Maximum Likelihood framework using RAxML v8.2.9 with the PROTGAMMALGF model of sequence evolution and 101 bootstraps. Asterics represent support values of 95 and above. A detailed method can be found in Kayal et al. 2018 BMC Evol. Biol. (https://doi.org/10.1186/s12862-018-1142-0). The full tree can be found at http://mmo.sb-roscoff.fr/jbrowseAmoebophrya/. Figure S2. SSU rDNA sequence identity (in percentage, relative to A25 and A120 compared to other species). Figure S3. Distribution of k-mer in A25 and A120 genomes. Figure S4. Classification of repeated elements in 3 Amoebophrya genomes (AT5, A25, and A120) using REPET. The x-axis represents the cumulated number of bases of repeated elements in the genome. Figure S5. Conserved motif of the putative splice leader (SL) in A25 and A120. Figure S6. Alignments of gene encoding the putative spliced leader (SL) gene in A25 and A120. Figure S7. Gene orientation change rate in 3 Amoebophrya genomes. Figure S8. Number of orthologs genes shared by selected taxa. Figure S9. Boxplot of the dN/dS ratios of orthologous genes between A25 and A120, calculated using the model average method (MA). Figure S10. Synteny dot-plot obtained by comparison between Amoebophrya A25 and AT5 genomes. Figure S11. Synteny dot-plot obtained by comparison between Amoebophrya A120 and AT5 genomes. Figure S12. Intron length distribution. Figure S13. GC content distribution. Figur [file 12915_2020_927_MOESM1_ESM.docx]

**Figure S1. Phylogeny of Alveolata.** Proteomes from 89 alveolates genomes and transcriptome assemblies from the MMETSP project (https://zenodo.org/record/257026/files/) were used to create orthologous groups using orthofinder v2.2 with the diamond BLAST similarity search. Single ortholog alignments were pruned using PhyloTreePruner v.1.0 (minimum taxa to keep 44 and support value 0.9) and realigned using mafft v7 and filtered with Gblocks v.0.91b (-b5=a -p=n ). Filtered alignments were concatenated using seqCat.pl and a phylogenetic tree was produced under Maximum Likelihood framework using RAxML v8.2.9 with the PROTGAMMALGF model of sequence evolution and 101 bootstraps. Asterics represent support values of 95 and above. A detailed method can be found in Kayal et al. 2018 BMC Evol. Biol. (10.1186/s12862-018-1142-0). The full tree can be found at http://mmo.sb-roscoff.fr/jbrowseAmoebophrya/.


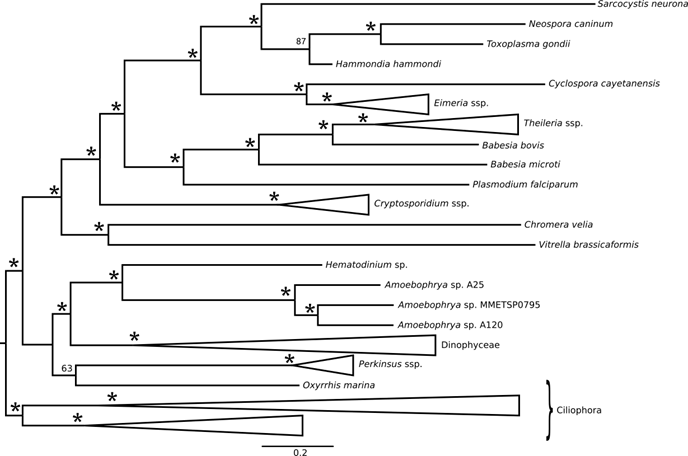


**List of species used:** *: For a given species, several strains have been merged.

| **Phylum** | **genus** | **sp** | **sample_nb_MMETSP** | **MMETSP** |
| --- | --- | --- | --- | --- |
| Apicomplexa | Babesia | bovis | Piroplasmadb | other |
| Apicomplexa | Babesia | microti | Piroplasmadb | other |
| Apicomplexa | Cryptosporidium | hominis | CryptoDB | other |
| Apicomplexa | Cryptosporidium | muris | CryptoDB | other |
| Apicomplexa | Cryptosporidium | parvum | CryptoDB | other |
| Apicomplexa | Cyclospora | cayetanensis | ToxoDB | other |
| Apicomplexa | Eimeria | brunetti | EnsemblProtists | other |
| Apicomplexa | Eimeria | tenella | EnsemblProtists | other |
| Apicomplexa | Hammondia | hammondi | EnsemblProtists | other |
| Apicomplexa | Lankesteria | abbottii | 1822 | MMETSP0372 |
| Apicomplexa | Neospora | caninum | ToxoDB | other |
| Apicomplexa | Plasmodinium | falciparum | PlasmoDB | other |
| Apicomplexa | Sarcocystis | neurona | ToxoDB | other |
| Apicomplexa | Theileria | annulata | Piroplasmadb | other |
| Apicomplexa | Theileria | equi | Piroplasmadb | other |
| Apicomplexa | Theileria | parva | Piroplasmadb | other |
| Apicomplexa | Toxoplasma | gondii | ToxoDB | other |
| Chromerids | Chromera | velia | CryptoDB | other |
| Chromerids | Vitrella | brassicaformis | CryptoDB | other |
| Ciliophora | Anophryoides | haemophila | 1960 | MMETSP1018* |
| Ciliophora | Anophryoides | haemophila | 2470 | MMETSP1019* |
| Ciliophora | Aristerostoma | sp | 1740 | MMETSP0125 |
| Ciliophora | Blepharisma | japonicum | 2217 | MMETSP1395 |
| Ciliophora | Climacostomum | virens | 2233 | MMETSP1397 |
| Ciliophora | Condylostoma | magnum | 1778 | MMETSP0210 |
| Ciliophora | Euplotes | crassus | 2220 | MMETSP1380 |
| Ciliophora | Euplotes | focardii | 2478 | MMETSP0205* |
| Ciliophora | Euplotes | focardii | 2479 | MMETSP0206* |
| Ciliophora | Euplotes | harpa | 1770 | MMETSP0213 |
| Ciliophora | Fabrea | salina | 2194 | MMETSP1345 |
| Ciliophora | Favella | taraikaensis | 1840 | MMETSP0434* |
| Ciliophora | Favella | taraikaensis | 1848 | MMETSP0436* |
| Ciliophora | Ichthyophthirius | multifilis | Ichthyophthirius Genome Database | other |
| Ciliophora | Litonotus | pictus | 1777 | MMETSP0209 |
| Ciliophora | Mesodinium | pulex | 1847 | MMETSP0467 |
| Ciliophora | Myrionecta | rubra | 2051 | MMETSP0798 |
| Ciliophora | Oxytricha | trifallax | McDonnell Genome Institute | other |
| Ciliophora | Paramecium | tetraurelia | ParameciumDB | other |
| Ciliophora | Platyophrya | macrostoma | 1732 | MMETSP0127 |
| Ciliophora | Protocruzia | adherens | 1776 | MMETSP0216 |
| Ciliophora | Pseudokeronopsis | sp | 1769 | MMETSP0211* |
| Ciliophora | Pseudokeronopsis | sp | 2218 | MMETSP1396* |
| Ciliophora | Strombidinopsis | acuminatum | 1731 | MMETSP0126 |
| Ciliophora | Strombidinopsis | sp | 1846 | MMETSP0463 |
| Ciliophora | Strombidium | inclinatum | 1763 | MMETSP0208 |
| Ciliophora | Strombidium | rassoulzadegani | 1841 | MMETSP0449 |
| Ciliophora | Tetrahymena | thermophila | Tetrahymena Genome Database | other |
| Ciliophora | Tiarina | fusus | 2034 | MMETSP0472 |
| Ciliophora | Uronema | sp | 1667 | MMETSP0018 |
| Dinophyceae | Akashiwo | sanguinea | 2497 | MMETSP0223_2 |
| Dinophyceae | Alexandrium | andersonii | 2266 | MMETSP1436 |
| Dinophyceae | Alexandrium | catenella | 2223 | MMETSP0790 |
| Dinophyceae | Alexandrium | fundyense | 1764 | MMETSP0196C* |
| Dinophyceae | Alexandrium | fundyense | 2515 | MMETSP0197* |
| Dinophyceae | Alexandrium | fundyense | 1805 | MMETSP0347* |
| Dinophyceae | Alexandrium | margalefi | 2036 | MMETSP0661 |
| Dinophyceae | Alexandrium | minutum | 1814 | MMETSP0328 |
| Dinophyceae | Alexandrium | monilatum | 1716 | MMETSP0093* |
| Dinophyceae | Alexandrium | monilatum | 1701 | MMETSP0095* |
| Dinophyceae | Alexandrium | monilatum | 1702 | MMETSP0096* |
| Dinophyceae | Alexandrium | monilatum | 1709 | MMETSP0097* |
| Dinophyceae | Alexandrium | tamarense | 1823 | MMETSP0378* |
| Dinophyceae | Alexandrium | tamarense | 1824 | MMETSP0380* |
| Dinophyceae | Alexandrium | tamarense | 1818 | MMETSP0382* |
| Dinophyceae | Alexandrium | tamarense | 1820 | MMETSP0384* |
| Dinophyceae | Amphidinium | carterae | 1792 | MMETSP0258* |
| Dinophyceae | Amphidinium | carterae | 1779 | MMETSP0259* |
| Dinophyceae | Amphidinium | carterae | 1819 | MMETSP0398C* |
| Dinophyceae | Amphidinium | carterae | 2481 | MMETSP0399* |
| Dinophyceae | Amphidinium | massartii | 2044 | MMETSP0689 |
| Dinophyceae | Azadinium | spinosum | 1864 | MMETSP1036* |
| Dinophyceae | Azadinium | spinosum | 1865 | MMETSP1037* |
| Dinophyceae | Azadinium | spinosum | 1866 | MMETSP1038* |
| Dinophyceae | Brandtodinium | nutriculum | 2287 | MMETSP1462 |
| Dinophyceae | Ceratium | fusus | 1911 | MMETSP1074* |
| Dinophyceae | Ceratium | fusus | 1883 | MMETSP1075* |
| Dinophyceae | Crypthecodinium | cohnii | 1810 | MMETSP0323* |
| Dinophyceae | Crypthecodinium | cohnii | 2189 | MMETSP0324* |
| Dinophyceae | Crypthecodinium | cohnii | 1811 | MMETSP0325* |
| Dinophyceae | Crypthecodinium | cohnii | 1812 | MMETSP0326* |
| Dinophyceae | Dinophysis | acuminata | 2050 | MMETSP0797 |
| Dinophyceae | Durinskia | baltica | 1730 | MMETSP0116* |
| Dinophyceae | Durinskia | baltica | 1717 | MMETSP0117* |
| Dinophyceae | Gambierdiscus | australes | 2030 | MMETSP0766 |
| Dinophyceae | Gonyaulax | spinifera | 2269 | MMETSP1439 |
| Dinophyceae | Gymnodinium | catenatum | 1665 | MMETSP0784 |
| Dinophyceae | Gyrodinium | dominans | 2134 | MMETSP1148 |
| Dinophyceae | Heterocapsa | arctica | 2271 | MMETSP1441 |
| Dinophyceae | Heterocapsa | rotundata | 1663 | MMETSP0503 |
| Dinophyceae | Heterocapsa | triquetra | 1852 | MMETSP0448 |
| Dinophyceae | Karenia | brevis | 1686 | MMETSP0027* |
| Dinophyceae | Karenia | brevis | 1687 | MMETSP0029* |
| Dinophyceae | Karenia | brevis | 1688 | MMETSP0030* |
| Dinophyceae | Karenia | brevis | 1689 | MMETSP0031* |
| Dinophyceae | Karenia | brevis | 1768 | MMETSP0201* |
| Dinophyceae | Karenia | brevis | 1762 | MMETSP0202* |
| Dinophyceae | Karenia | brevis | 2054 | MMETSP0527* |
| Dinophyceae | Karenia | brevis | 2061 | MMETSP0528* |
| Dinophyceae | Karenia | brevis | 2070 | MMETSP0573* |
| Dinophyceae | Karenia | brevis | 2079 | MMETSP0574* |
| Dinophyceae | Karenia | brevis | 1966 | MMETSP0648* |
| Dinophyceae | Karenia | brevis | 2035 | MMETSP0649* |
| Dinophyceae | Karlodinium | micrum | 1947 | MMETSP1015* |
| Dinophyceae | Karlodinium | micrum | 1953 | MMETSP1016* |
| Dinophyceae | Karlodinium | micrum | 1954 | MMETSP1017* |
| Dinophyceae | Kryptoperidinium | foliaceum | 1718 | MMETSP0118* |
| Dinophyceae | Kryptoperidinium | foliaceum | 1721 | MMETSP0119* |
| Dinophyceae | Kryptoperidinium | foliaceum | 1722 | MMETSP0120* |
| Dinophyceae | Kryptoperidinium | foliaceum | 1719 | MMETSP0121* |
| Dinophyceae | Lessardia | elongata | 2133 | MMETSP1147 |
| Dinophyceae | Lingulodinium | polyedra | 1860 | MMETSP1032* |
| Dinophyceae | Lingulodinium | polyedra | 1861 | MMETSP1033* |
| Dinophyceae | Lingulodinium | polyedra | 1862 | MMETSP1034* |
| Dinophyceae | Lingulodinium | polyedra | 1863 | MMETSP1035* |
| Dinophyceae | Noctiluca | scintillans | 1791 | MMETSP0253 |
| Dinophyceae | Oxyrrhis | marina | 1697 | MMETSP0044* |
| Dinophyceae | Oxyrrhis | marina | 1842 | MMETSP0451_2C* |
| Dinophyceae | Oxyrrhis | marina | 2509 | MMETSP0452_2* |
| Dinophyceae | Oxyrrhis | marina | 1843 | MMETSP0468* |
| Dinophyceae | Oxyrrhis | marina | 1844 | MMETSP0469* |
| Dinophyceae | Oxyrrhis | marina | 1845 | MMETSP0470* |
| Dinophyceae | Oxyrrhis | marina | 2033 | MMETSP0471* |
| Dinophyceae | Oxyrrhis | marina | 2242 | MMETSP1424* |
| Dinophyceae | Oxyrrhis | marina | 2243 | MMETSP1425* |
| Dinophyceae | Oxyrrhis | marina | 2256 | MMETSP1426* |
| Dinophyceae | Pelagodinium | beii | 2501 | MMETSP1338 |
| Dinophyceae | Peridinium | aciculiferum | 1816 | MMETSP0370* |
| Dinophyceae | Peridinium | aciculiferum | 1817 | MMETSP0371* |
| Dinophyceae | Polarella | glacialis | 1775 | MMETSP0227* |
| Dinophyceae | Polarella | glacialis | 2270 | MMETSP1440* |
| Dinophyceae | Prorocentrum | micans | 1789 | MMETSP0251 |
| Dinophyceae | Prorocentrum | minimum | 2475 | MMETSP0053* |
| Dinophyceae | Prorocentrum | minimum | 2476 | MMETSP0055* |
| Dinophyceae | Prorocentrum | minimum | 2511 | MMETSP0056* |
| Dinophyceae | Prorocentrum | minimum | 2477 | MMETSP0057* |
| Dinophyceae | Prorocentrum | minimum | 1780 | MMETSP0267* |
| Dinophyceae | Prorocentrum | minimum | 1783 | MMETSP0268* |
| Dinophyceae | Prorocentrum | minimum | 1784 | MMETSP0269* |
| Dinophyceae | Prorocentrum | reticulatum | 1773 | MMETSP0228 |
| Dinophyceae | Pyrocystis | lunula | 1774 | MMETSP0229 |
| Dinophyceae | Pyrodinium | bahamense | 2049 | MMETSP0796 |
| Dinophyceae | Scrippsiella | hangoei | 1806 | MMETSP0359* |
| Dinophyceae | Scrippsiella | hangoei | 1807 | MMETSP0360* |
| Dinophyceae | Scrippsiella | hangoei | 1808 | MMETSP0361* |
| Dinophyceae | Scrippsiella | hangoei-like | 1825 | MMETSP0367* |
| Dinophyceae | Scrippsiella | hangoei-like | 1826 | MMETSP0368* |
| Dinophyceae | Scrippsiella | hangoei-like | 1827 | MMETSP0369* |
| Dinophyceae | Scrippsiella | trochoidea | 1781 | MMETSP0270* |
| Dinophyceae | Scrippsiella | trochoidea | 1782 | MMETSP0271* |
| Dinophyceae | Scrippsiella | trochoidea | 1804 | MMETSP0272* |
| Dinophyceae | Symbiodinium | kawagutii | 1739 | MMETSP0132_2C* |
| Dinophyceae | Symbiodinium | kawagutii | 2514 | MMETSP0133_2* |
| Dinophyceae | Symbiodinium | kawagutii | 2518 | MMETSP0134_2* |
| Dinophyceae | Symbiodinium | kawagutii | 2516 | MMETSP0135_2* |
| Dinophyceae | Symbiodinium | microadiaticum | Reef Genomics | other |
| Dinophyceae | Symbiodinium | minutum | Marine Genomics Unit 2015 | other |
| Dinophyceae | Symbiodinium | sp | 2187 | MMETSP1110 |
| Dinophyceae | Symbiodinium | sp | 1885 | MMETSP1115* |
| Dinophyceae | Symbiodinium | sp | 1886 | MMETSP1116* |
| Dinophyceae | Symbiodinium | sp | 1887 | MMETSP1117* |
| Dinophyceae | Symbiodinium | sp | 1897 | MMETSP1122* |
| Dinophyceae | Symbiodinium | sp | 1898 | MMETSP1123* |
| Dinophyceae | Symbiodinium | sp | 1903 | MMETSP1124* |
| Dinophyceae | Symbiodinium | sp | 1904 | MMETSP1125* |
| Dinophyceae | Symbiodinium | sp | 2193 | MMETSP1374 |
| Dinophyceae | Thoracosphaera | heimii | 1772 | MMETSP0225 |
| Dinophyceae | Togula | jolla | 1771 | MMETSP0224 |
| Perkinsea | Parvilucifera | infectans |  | other |
| Perkinsea | Parvilucifera | rostrata |  | other |
| Perkinsea | Perkinsus | chesapeaki | 2005 | MMETSP0924C* |
| Perkinsea | Perkinsus | chesapeaki | 2485 | MMETSP0925* |
| Perkinsea | Perkinsus | marinus | EnsemblProtists | other |
| Syndiniales | Amoebophrya | A25 |  | other |
| Syndiniales | Amoebophrya | A120 |  | other |
| Syndiniales | Amoebophrya | sp | 2046 | MMETSP0795 |
| Syndiniales | Hematodinium |  |  | other |

**Figure S2. SSU rDNA sequence identity (in percentage, relative to A25 and A120 compared to other species)**


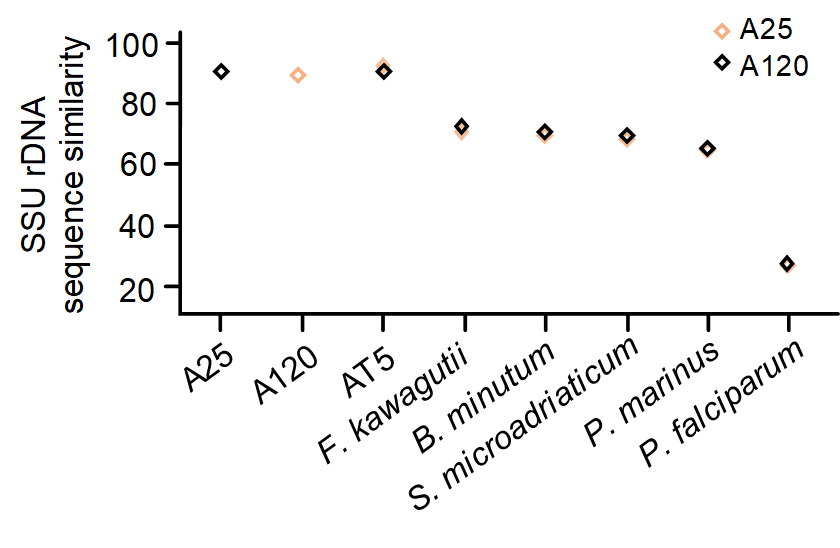


**Figure S3. Distribution of k-mer in A25 and A120 genomes.**

Analysis of k-mer from Illumina 100 bp paired-end genomic reads of both *Amoebophrya,* including the genome size estimation. **A**: A25, **B**: A120.


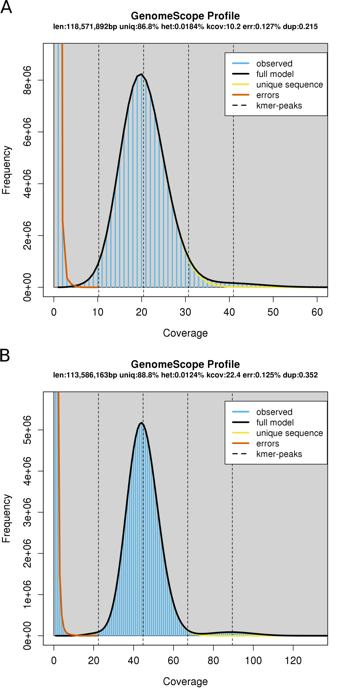


**Figure S4.** Classification of repeated elements in 3 *Amoebophrya* genomes (AT5, A25, and A120) using REPET. The x-axis represents the cumulated number of bases of repeated elements in the genome.


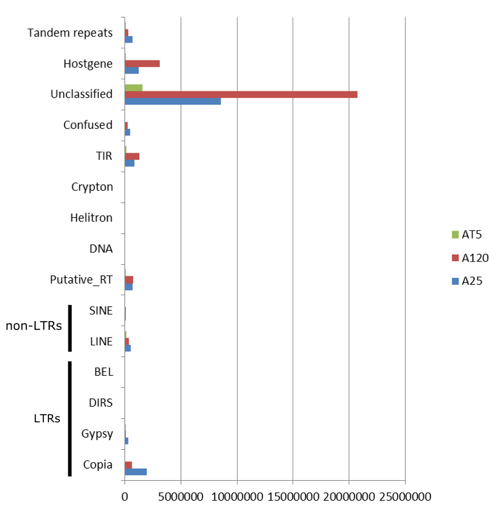


**Figure S5. Conserved motif of the putative splice leader (SL) in A25 and A120.**


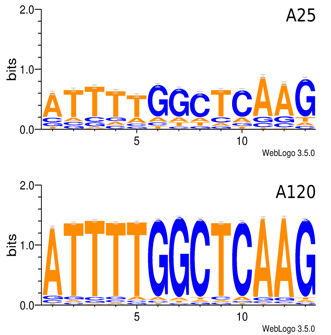


**Figure S6. Alignments of gene encoding the putative *spliced leader* (SL) gene in A25 and A120.**

A25 and A120 SL encoding genes are compared to other sequences from *Amoebophrya* (Amo-XX) and *Karenia* spp. (*K. mikimotoi* and *K. brevis*) previously published by Zhang et al. (2011).

**
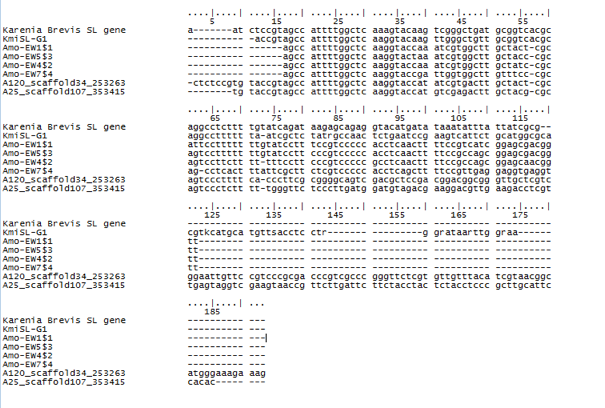
**

**Figure S7. Gene orientation change rate in 3 *Amoebophrya* genomes.**

Number of changes in gene orientation in *Amoebophrya* A25 and A120 were compared to *Amoebophrya* AT5 (AT5), *Fugacium kawagutii* (Fkav), *Symbiodinium microadriaticum* (Smic) and *Breviolum minutum* (Bmin). Gene orientation was computed using a non-overlapping 10 genes sliding window.

**
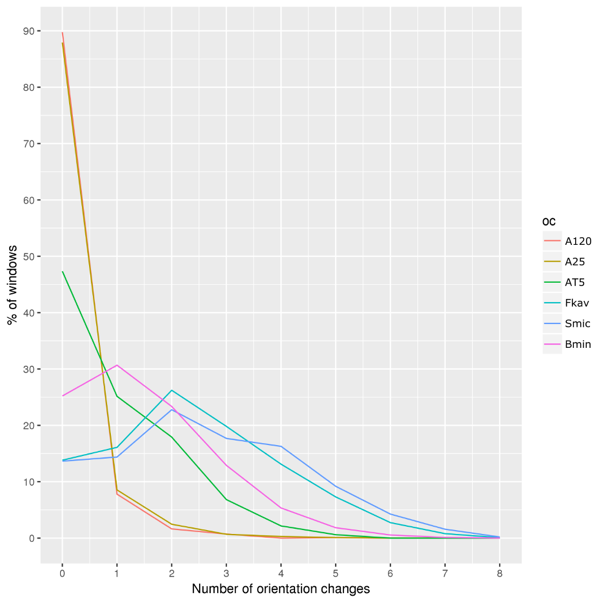
**

**Figure S8. Number of orthologs genes shared by selected taxa.**

The bars show the number of clusters of orthologous genes (OGs) among selected species (grey dots). Species used: *Amoebophrya* A25 (A25), *Amoebophrya* A120 (A120), *F. kawagutii* (Fkaw), *S. microadriaticum* (Smic), and *B. minutum* (Bmin), *P. marinus* (Pmar), *P. falciparum* (Pfal), *T. gondii* (Tgon), *T. brucei* (Tbru), *L. major* (Lmar), *T. equi* (Tequ), *C. velia* (Cvel), *V. brassicaformis* (Vbra) and *C. parvum* (Cpar). Selected taxa shown in the figure: Syndiniales (orange), Euglenozoa (blue), chromerids (burgundy), Symbiodiniaceae (dark green), Apicomplexa (red), dinoflagellates (magenta), Alveolata (light green), Eukaryota (light grey).


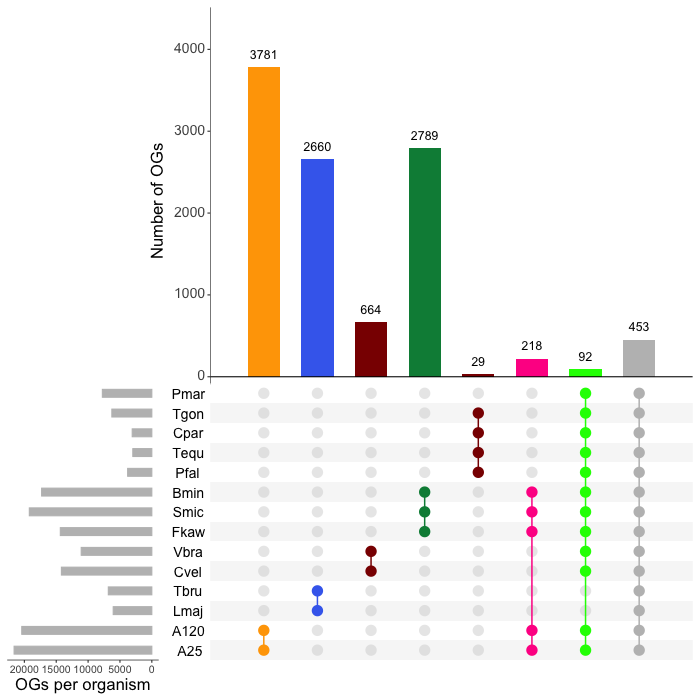


**Figure S9.** Boxplot of the dN/dS ratios of orthologous genes between A25 and A120, calculated using the model average method (MA).


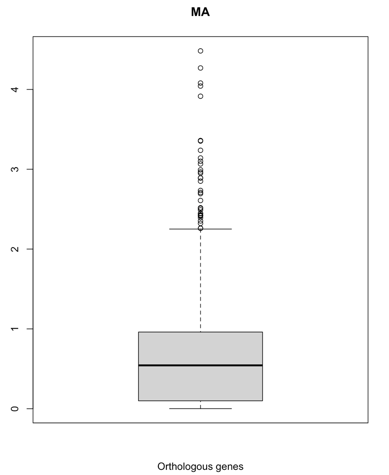


**Figure S10. Synteny dot-plot obtained by comparison between *Amoebophrya* A25 and AT5 genomes**

Dot-plot of synteny between the longest scaffolds for each of the *Amoebophrya* AT5 and A25 genomes. The 100 scaffolds (AT5) and the 100 scaffolds (A25) are shown on the x and y axes, respectively. For each genome, genes are sorted by their rank on the scaffolds. Each blue point represents a pair of orthologous genes defined by BRH.


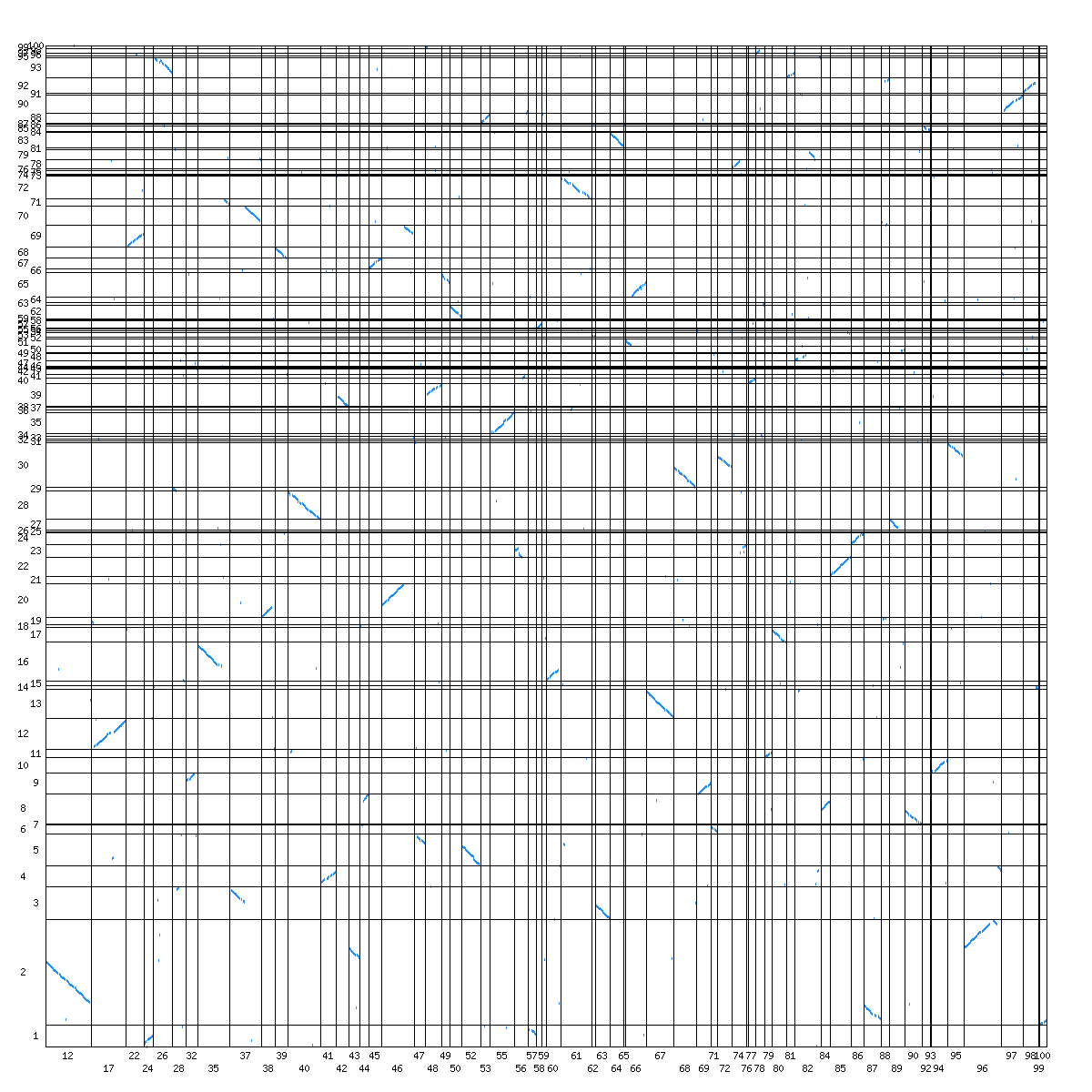


**Figure S11. Synteny dot-plot obtained by comparison between *Amoebophrya* A120 and AT5 genomes.**

Dot-plot of synteny between the longest scaffolds for each of the *Amoebophrya* AT5 and A120 genomes. The 100 scaffolds (AT5) and the 100 scaffolds (A120) are shown on the x and y axes, respectively. For each genome, genes are sorted by their rank on the scaffolds. Each blue point represents a pair of orthologous genes defined by BRH.


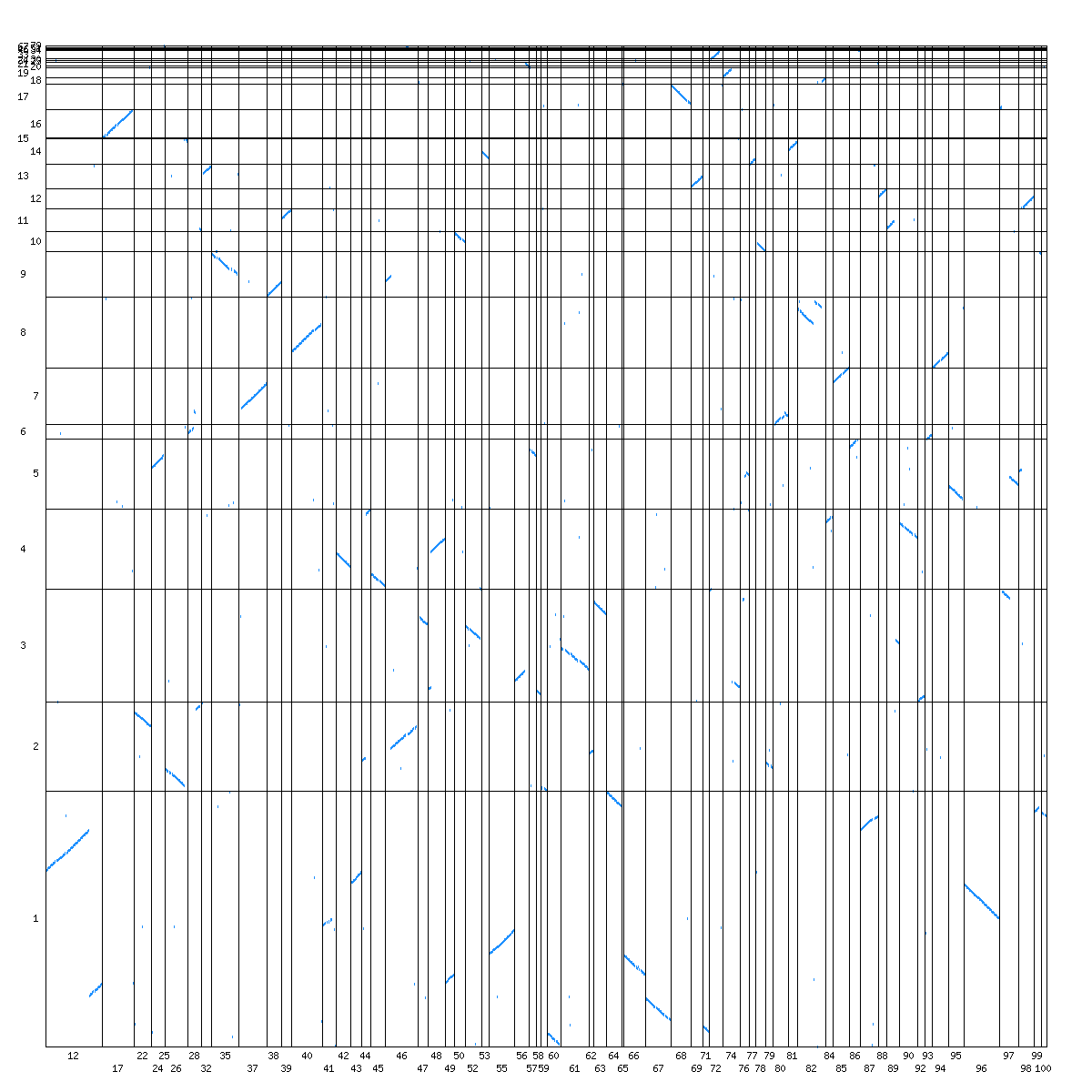


**Figure S12. Intron length distribution.**

Length distribution (in percent) of canonical and non-canonical (NCIs) introns for the *Amoebophrya* A25 (A) and A120 (B) genomes.


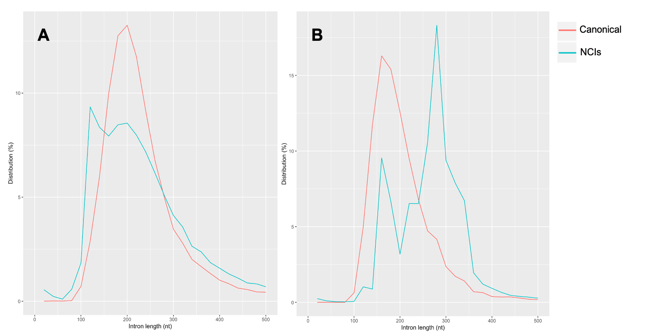


**Figure S13. GC content distribution.**

Distribution (in percent) of GC content in canonical and non-canonical (NCIs) introns for the *Amoebophrya* A25 (A) and A120 (B) genomes.


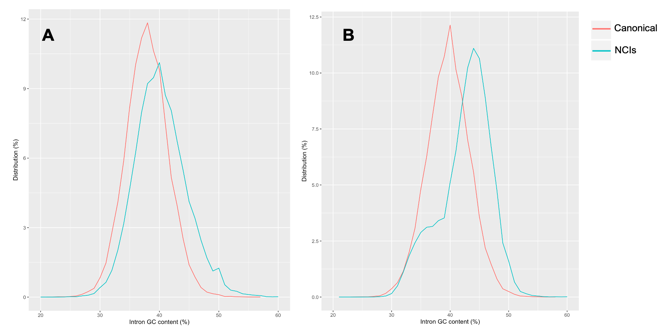


**Figure S14. Multiple alignments of U2 snRNAs.**

Multiple alignment of U2 snRNAs of *Amoebophrya* A25 and A120 (A25-U2A_Dinospore, A25-U2B_T12, A25-U2C_T24, A25-U2D_T30, A25-U2E_T36, A25-U2F_T42, A25-U2G_T6 and A120-U2A_T12, A120-U2B_T18, A120-U2C_T24, A120-U2D_T30, A120-U2E_T6) with *P. falciparum*, *B. minutum* and *H. sapiens* U2 snRNAs (Pf-u2snRNA, Sm-u2snRNA and Hs-u2snRNA)


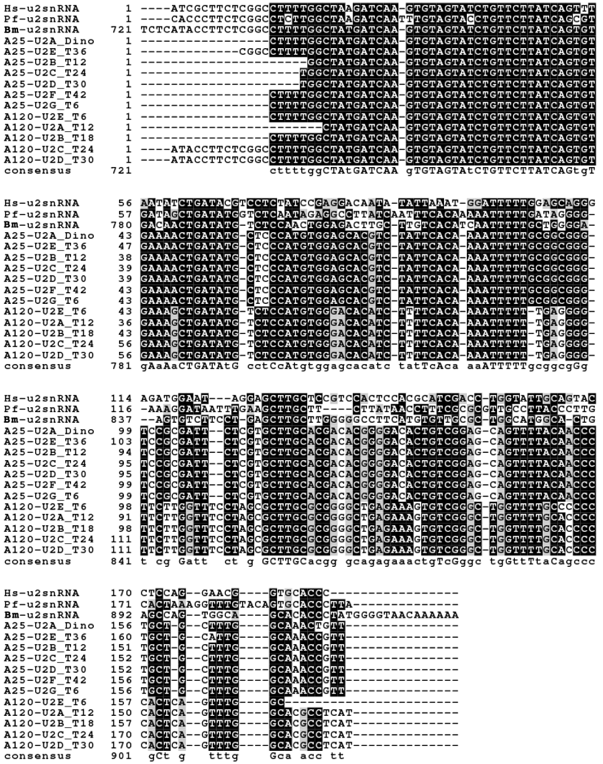


**Figure S15. Multiple alignments of U4 snRNAs.**

Multiple alignment of U4 snRNAs of *Amoebophrya* A25 and A120 (A25-U4_Dinospore and A120-U4A_Dinospore, A120-U4B_T18, A120-U4C_T30, A120-U4D_T36) with *P. falciparum*, *B. minutum* and *H. sapiens* U4 snRNAs (Pf-u4snRNA, Sm-u4snRNA and Hs-u4snRNA)


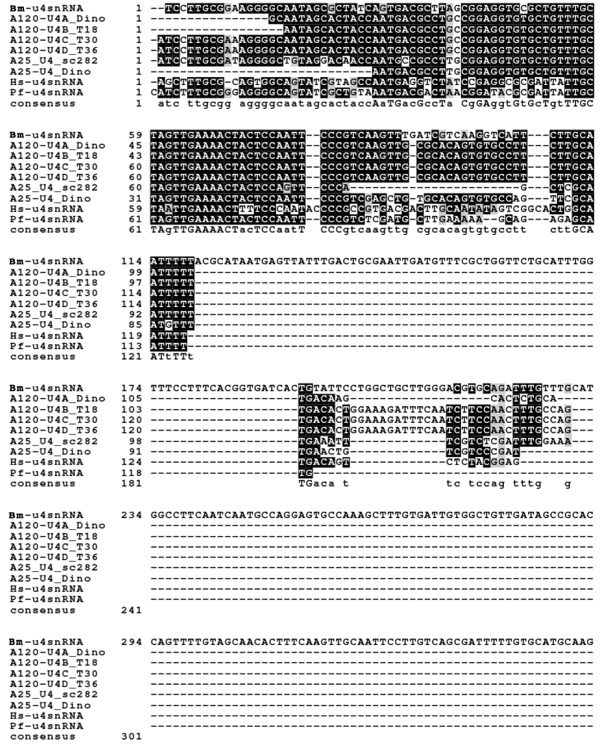


**Figure S16. Multiple alignments of U5 snRNAs.**

Multiple alignment of U5 snRNAs of Amoebophrya A120 (A120-U5A_T30 and A120-U5B_T36) with *P. falciparum*, *B minutum* and *H. sapiens* U4 snRNAs (Pf-u5snRNA, Sm-u5snRNA and Hs-u5snRNA)

**
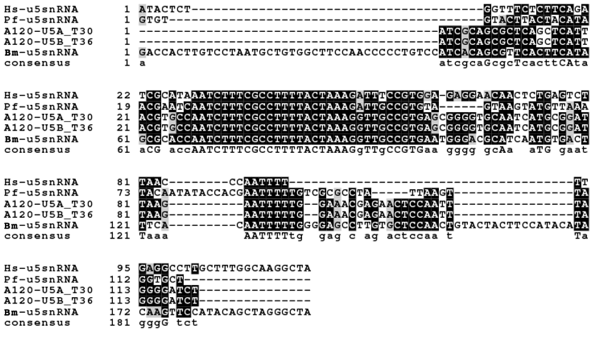
**

**Figure S17. Multiple alignments of U6 snRNAs.**

Multiple alignment of U6 snRNAs of *Amoebophrya* A25 and A120 with *P. falciparum*, *B. minutum* and *H. sapiens* U6 snRNAs (Pf-u6snRNA, Sm-u6snRNA and Hs-u6snRNA).


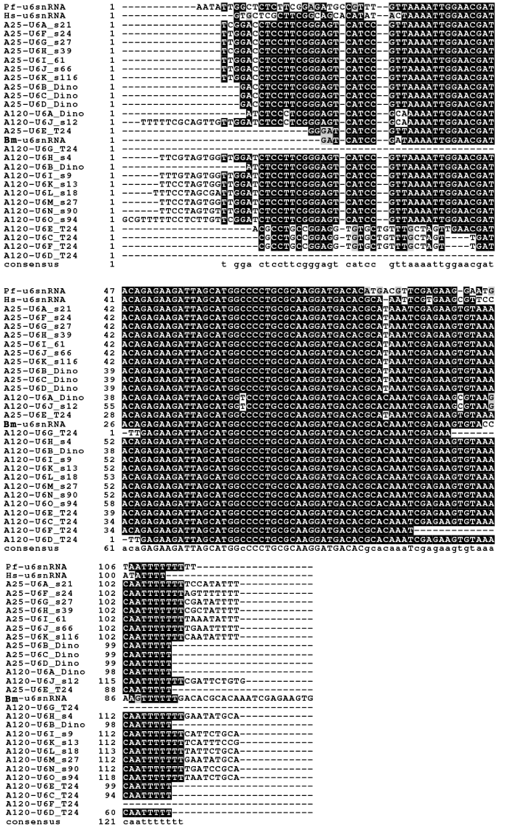


**Figure S18. Secondary structure of *Amoebophrya* snRNA.**

U2, U4, U5 and U6 secondary structure of *H. sapiens*, A25 and A120 (lack of A25 U5 snRNA). A is U2 snRNAs, B is U4 snRNA, C is U5 snRNA and D is U6 snRNA.

**
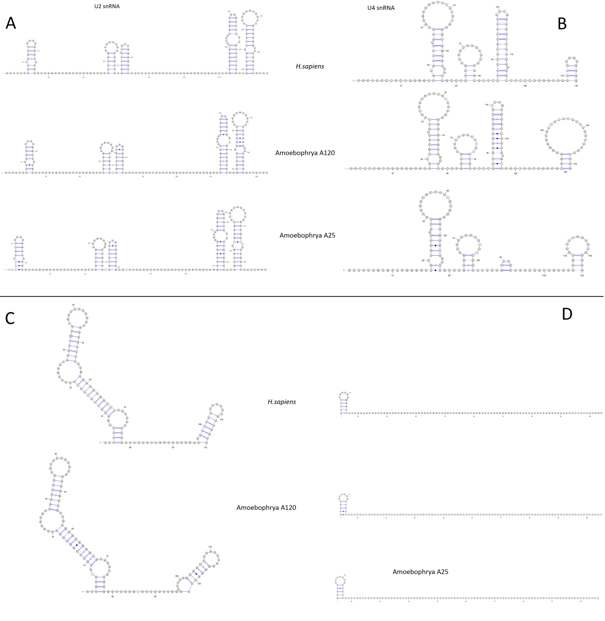
**

**Figure S19. Example of introner elements (IEs) in *Amoebophrya*.**

Direct repeats of 5 bp are shown in blue. Inverted repeats (red) are at the introner element ends. Squares are the exon sequence border prediction.


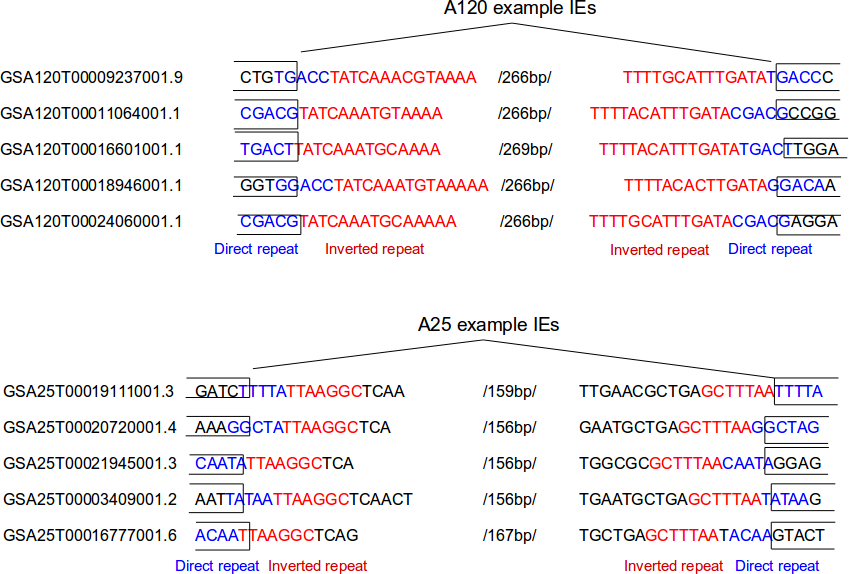


**Figure S20. Distribution the direct repeats with size ranging between 3 and 8 nucleotides in A25.**


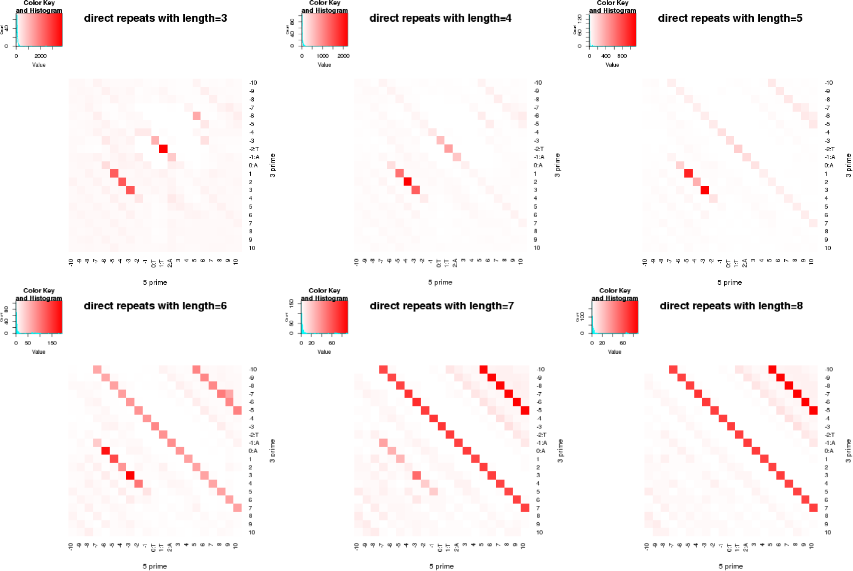


**Figure S21. Distribution of the direct repeats with size ranging between 3 and 8 nucleotides in A120.**


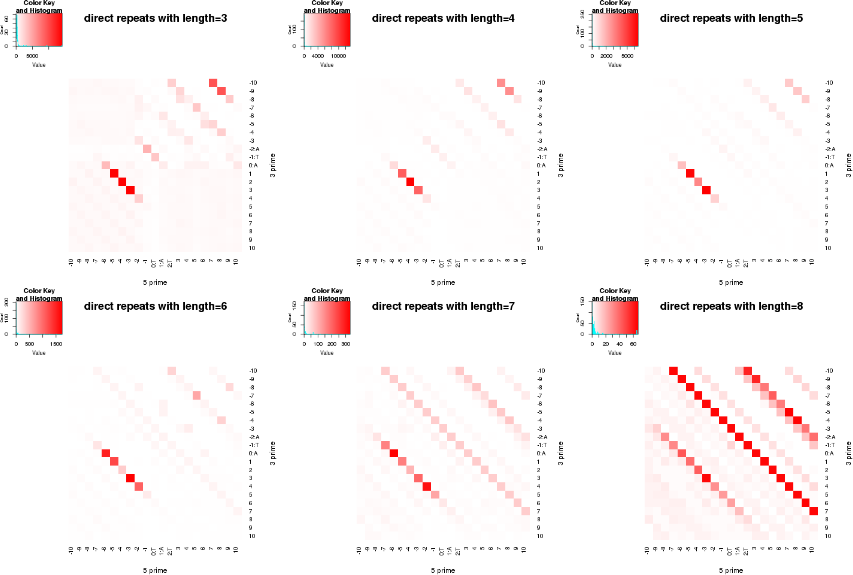


**Figure S22. Composition of direct repeats in introners elements.**

The diversity in composition of the three (a, b, c) most abundant of direct repeats in introner elements in A25 (up) and A120 (down).


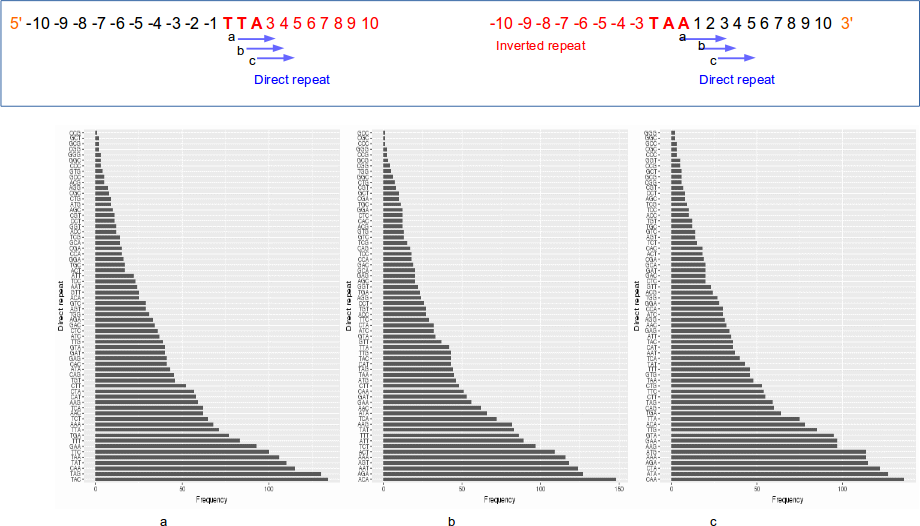


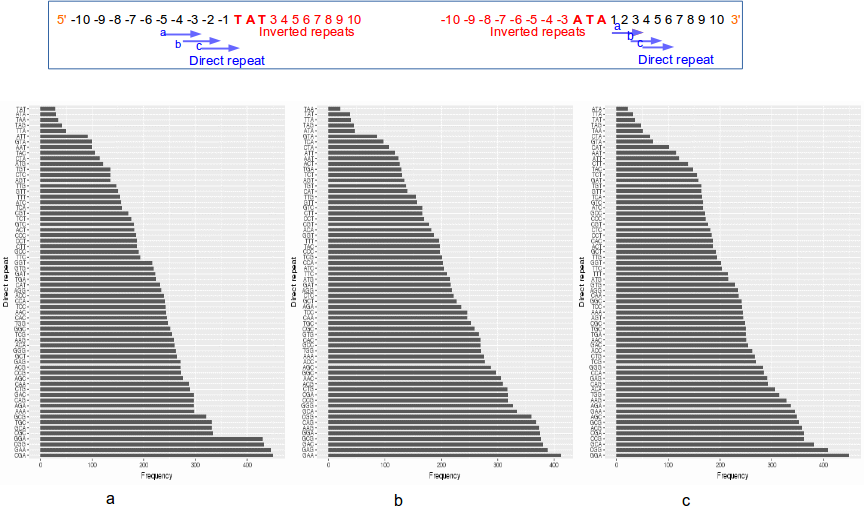


**Figure S23. Terminal inverted repeat locations around the splicing sites in A25 and A120.**

The position of inverted repeats according to the location of the splice sites in A25 and A120. Left, the inverted repeats of A120 are located at 1-5 the nucleotides upstream and downstream of the splice sites. Right, the inverted repeats of A25 are located at the 1-6 nucleotides in upstream and downstream of the splice sites.


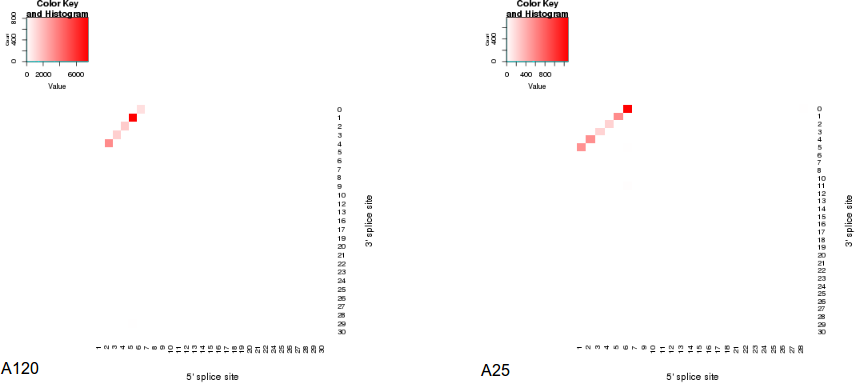


**Figure S24. The flowchart for the *in silico* search of introner elements.**


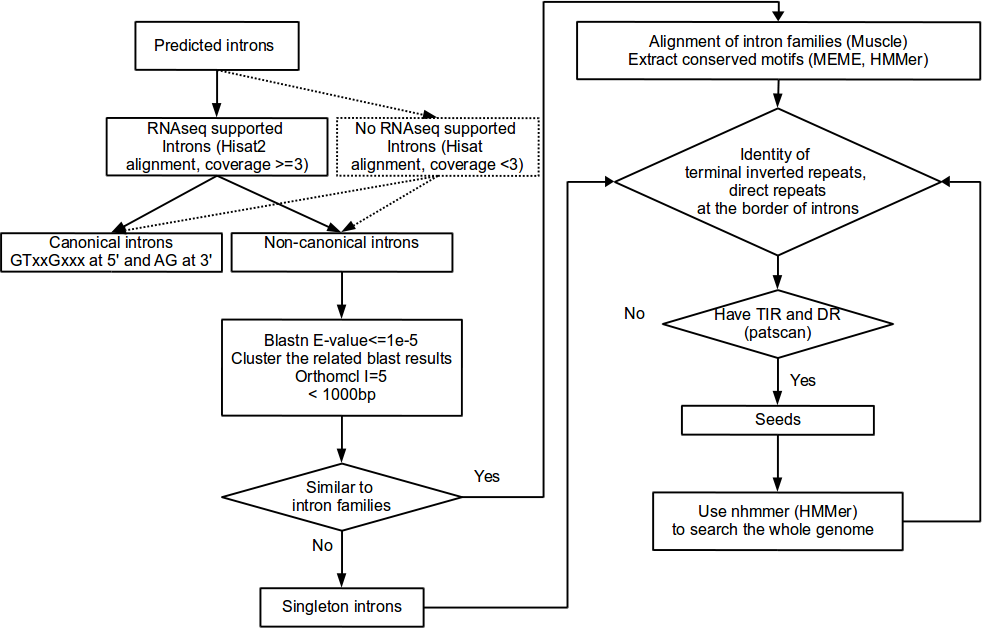


**Figure S25.** Hierarchical clustering analysis (pairwise similarity and OrthoMCL) of all intron families and of the inverted repeats in A25 and A120.


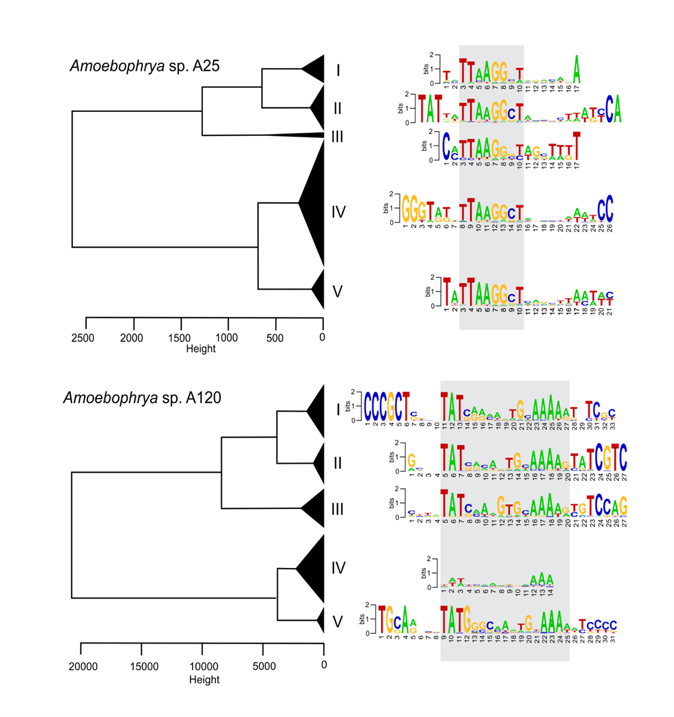


**Figure S26. Percentage of genes with assigned functions in relation with introns composition.**

Bar plot of the percentage of genes with or without assigned function for the three categories of genes defined by their composition in introns in *Amoebophrya* A25 and A120 (percentage of total spliced genes per species).

**
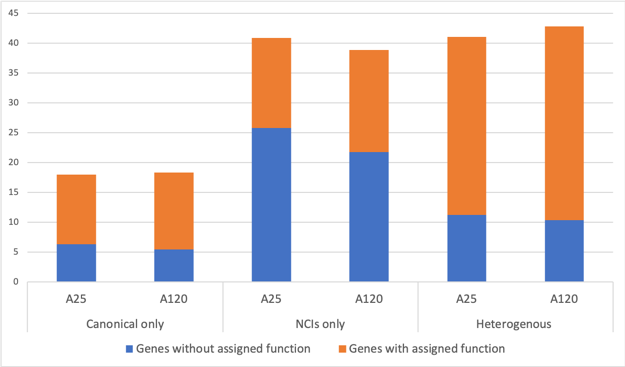
**

**Figure S27. Difference in the proportion of IEs-containing-genes compared to their KEGG assignment in A25 and A120.**

Integration of IEs is statistically different in genes involved in translation (A25), and coding for ribosomal proteins (A120 and A25).

A25


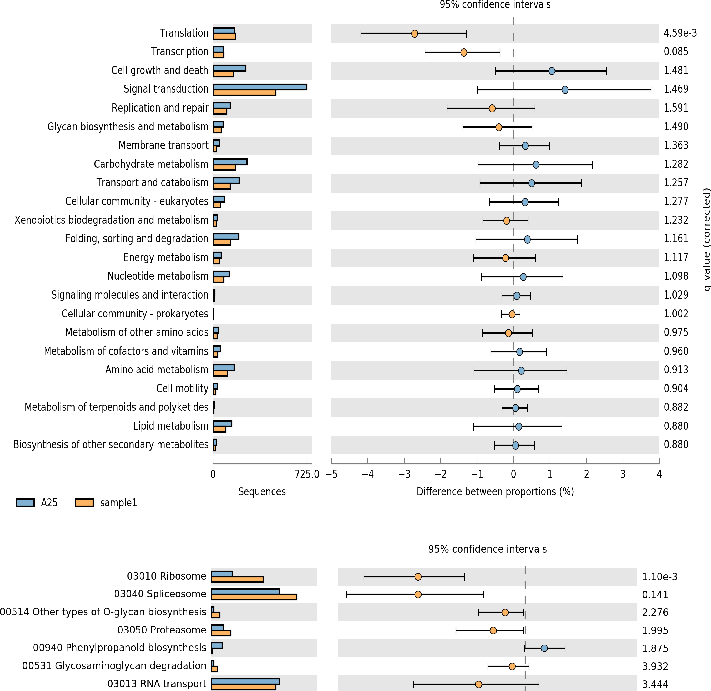


| A120 |
| --- |
| 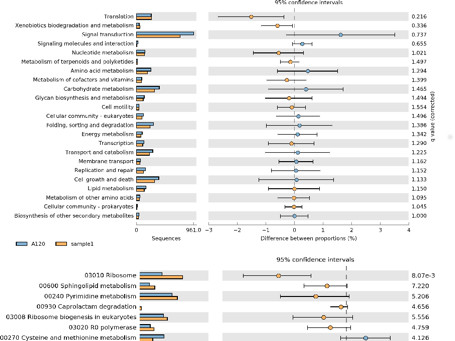 |
|  |
|  |

**Figure S28. Distribution of conserved introns.**

Violin plot distribution of the ratio of conserved intron based on the level of amino acid level identity of aligned orthologous genes. Percent identity is shown on the x axis. A diamond represents the average ratio of conserved introns for each violin plot. The minimum alignment length for each orthologous pair was >80%.


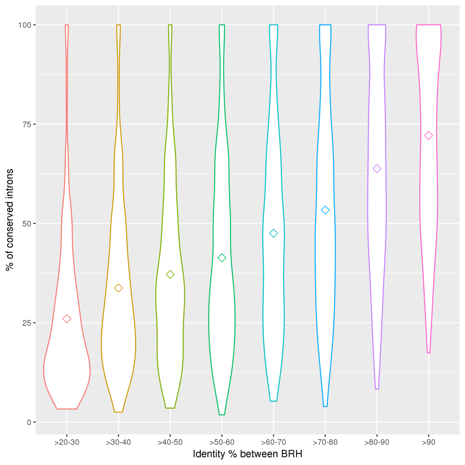


**Table S1. RCC number, date and site of isolation of strains considered in this study.**

| **Strain** | **RCC number** | **Date and site of isolation** |
| --- | --- | --- |
| *Scrippsiella acuminata* (ST147)* | RCC1627 | 2005 from sediment |
| *Heterocapsa triquetra* (HT150) | RCC3596 | July 6th 2007 |
| A25** | RCC4383 | 15th of June 2009 |
| A120*** | RCC4398 | 13th of June 2011 |

* previously known as *S. trochoidea*, Kretschmann et al. (2015)

http://dx.doi.org/10.11646/phytotaxa.220.3.3

** First isolated in *Scrippsiella acuminata*

*** First isolated in *Heterocapsa triquetra*, then maintained in *Scrippsiella acuminata*

Table S2. Metrics of Nanopore runs for the two *Amoebophrya* strains.

|  | **A25** | **A120** |
| --- | --- | --- |
| Number of runs | 2 | 3 |
| Cumulative size (nt) | 2,534,247,679 | 14,022,482,812 |
| Average size (nt) | 15,244 | 9,344 |
| N50 (nt) | 19,456 | 14,562 |

Table S3. Search for pathways involved in plastidial functions that are entirely independent of plastid-encoded gene content.

NA: absent (lost?), N: no signal, #: signal peptide using SignalPv5 and TargetPv2 for Plants

| **Pathway** | **Name of the protein** | **Gene_ID** | **A120** | **A25** | **Signal peptide#** | **Phylogeny_pos** |
| --- | --- | --- | --- | --- | --- | --- |
| **Calvin cycle** | ribulose-bisphosphate carboxylase (4.1.1.39) | Rubisco | NA | NA | NA | NA |
| specific enzymes | fructose-1,6-bisphosphatase (3.1.3.11) | FBP | NA | NA | NA | NA |
|  | fructose-bisphosphate aldolase (4.1.2.13) | FBA | NA | NA | NA | NA |
|  | sedoheptulose-1,7-bisphosphatase (3.1.3.37) | SBP | GSA120T00003385001 | GSA25T00004323001 | N | group with SBP. This protein however may have been retained for other function cellular function as suggested by Petersen et al. (2014) |
|  | phosphoribulokinase (2.7.1.19) | PRK | NA | NA | NA | NA |
|  |  |  |  |  |  |  |
| **Glycolysis/Gluconeogenesis** | fructose-1,6-bisphosphatase (3.1.3.11) | FBP | GSA120T00014866001 | GSA25T00008308001 | N | group with FBP |
| * in common with Calvin cycle | fructose-bisphosphate aldolase (4.1.2.13) | ALDO | GSA120T00011127001 | GSA25T00000645001 | N | no specificity |
|  | fructose-bisphosphate aldolase (4.1.2.13) | ALDO |  | GSA25T00006403001 | N | long branch |
|  | glyceraldehyde-3-phosphate dehydrogenase (1.2.1.12)* | GAPDH | GSA120T00017876001 | GSA25T00004712001 | N | heterotroph clade |
|  | glyceraldehyde-3-phosphate dehydrogenase (1.2.1.12)* | GAPDH | GSA120T00020038001 | GSA25T00004810001 | N | heterotroph clade |
|  | phosphoglycerate kinase (2.7.2.3)* | PGK | GSA120T00014194001 | GSA25T00018513001 | N | heterotroph clade |
|  | phosphoglycerate mutase (5.4.2.11) | PGAM | GSA120T00015923001 | GSA25T00025967001 | N | heterotroph clade |
|  | enolase (4.2.1.11) | ENO | GSA120T00018016001 | GSA25T00002225001 | N | no specificity |
|  | pyruvate kinase (2.7.1.40) | PYK | GSA120T00004937001 | GSA25T00009723001 | N |  |
|  | pyruvate kinase (2.7.1.40) | PYK | GSA120T00005726001 | GSA25T00026997001 | N |  |
|  | pyruvate carboxylase (6.4.1.1) | PC | GSA120T00025777001 | GSA25T00008414001 | N | long branch |
|  |  |  |  |  |  |  |
| **Heme synthesis** | 5-aminolevulinate synthase | ALAS | GSA120T00021910001 | GSA25T00007028001 | N/mt | no specificity |
|  | Glutamyl-tRNA reductase | HemA | NA | NA |  | NA |
|  | Glutamate-1-semialdehyde 2,1-aminomutase | HemL | GSA120T00012860001 | GSA25T00001948001 | N | no specificity |
|  | Porphobilinogen synthase | HemB | GSA120T00007389001 | NA | N | group with cytosolic sequences |
|  | Hydroxymethylbilane synthase | HemC | GSA120T00008083001 | NA | N | group with cytosolic sequences |
|  | Uroporphyrinogen-III synthase | HemD | GSA120T00022736001 | GSA25T00003669001 | N | no specificity |
|  | Uroporphyrinogen decarboxylase | HemE | GSA120T00023402001 | NA | N | group with cytosolic sequences? |
|  | Oxygen-independent coproporphyrinogen III oxidase | HemN | NA | NA | N |  |
|  | Oxygen-dependent protoporphyrinogen oxidase | HemY | GSA120T00008260001 | GSA25T00001129001 | N | heterotroph clade |
|  | Ferrochelatase | HemH | NA | NA |  | NA |
|  | COX10, heme A:farnesyltransferase cytochrome c oxidase assembly factor | COX10 | GSA120T00022216001 | GSA25T00010968001 | N | no specificity |
|  | COX15, cytochrome c oxidase assembly homolog | COX15 | GSA120T00017165001 | GSA25T00017470001 | N | no specificity |
|  | Holocytochrome-c synthase ; cytochrome C | HCCS | GSA120T00007491001 | GSA25T00023177001 | N | no specificity |
|  |  |  |  |  |  |  |
| **t-RNA synthetase** | Alanine--tRNA ligase, cytoplasmic | AARS | GSA120T00006257001 | GSA25T00026864001 | N |  |
|  | Cysteine--tRNA ligase, cytoplasmic | CARS | GSA120T00019489001 | GSA25T00005346001 | mt/N |  |
|  | Aspartate--tRNA ligase, cytoplasmic | DARS | GSA120T00018038001 | GSA25T00002198001 | N |  |
|  | Glutamate--tRNA ligase, cytoplasmic | EARS | GSA120T00001932001 | GSA25T00010884001 | N |  |
|  | Glutamate--tRNA ligase, cytoplasmic | EARS | GSA120T00001941001 | NA | N |  |
|  | Glutamine--tRNA ligase, cytoplasmic | QARS | GSA120T00000834001 | GSA25T00021216001 | N |  |
|  | Phenylalanine--tRNA ligase, mitochondrial | FARS2 | GSA120T00011125001 | GSA25T00000647001 | mt/N |  |
|  | Phenylalanine--tRNA ligase alpha subunit | FARSA | GSA120T00023523001 | GSA25T00011373001 | N |  |
|  | Phenylalanine--tRNA ligase beta subunit | FARSB | GSA120T00017526001 | GSA25T00001916001 | N |  |
|  | Glycine--tRNA ligase, cytoplasmic | GARS | GSA120T00017655001 | GSA25T00005550001 | N/cp |  |
|  | Histidine--tRNA ligase, cytoplasmic | HARS | GSA120T00003631001 | GSA25T00024405001 | N |  |
|  | Isoleucine--tRNA ligase, cytoplasmic | IARS | GSA120T00024080001 | GSA25T00020070001 | N/cp |  |
|  | Lysine--tRNA ligase, cytoplasmic | KARS | GSA120T00010372001 | GSA25T00017931001 | mt/N |  |
|  | Leucine--tRNA ligase, cytoplasmic | LARS | GSA120T00001238001 | GSA25T00008624001 | N |  |
|  | Methionine--tRNA ligase, cytoplasmic | MARS | GSA120T00005652001 | GSA25T00018977001 | N |  |
|  | Asparagine--tRNA ligase, cytoplasmic | NARS | GSA120T00013280001 | GSA25T00025378001 | N |  |
|  | Proline--tRNA ligase, cytoplasmic | PARS | GSA120T00004947001 | GSA25T00005837001 | N/mt |  |
|  | Proline--tRNA ligase, mitochondrial | PARS2 | GSA120T00025685001 | GSA25T00016457001 | N |  |
|  | Arginine--tRNA ligase, cytoplasmic | RARS | GSA120T00007572001 | GSA25T00013311001 | N |  |
|  | Serine--tRNA ligase, cytoplasmic | SARS | GSA120T00023938001 | GSA25T00019413001 | N |  |
|  | Serine--tRNA ligase, cytoplasmic | SARS | GSA120T00020461001 | GSA25T00001048001 | N |  |
|  | Threonine--tRNA ligase, cytoplasmic | TARS | GSA120T00008438001 | GSA25T00025461001 | N |  |
|  | Threonine--tRNA ligase, cytoplasmic | TARS | GSA120T00008439001 | NA | N |  |
|  | Valine--tRNA ligase, cytoplasmic | VARS | GSA120T00014516001 | GSA25T00010092001 | N |  |
|  | Tryptophan--tRNA ligase, cytoplasmic | WARS | GSA120T00013453001 | GSA25T00026783001 | N/mt |  |
|  | Tryptophan--tRNA ligase, cytoplasmic | WARS |  | GSA25T00027686001 | N |  |
|  | Tyrosine--tRNA ligase, cytoplasmic | YARS | GSA120T00015394001 | GSA25T00012510001 | mt/N |  |
|  |  |  |  |  |  |  |
| **MinD/MinE** |  | MinD | NA | NA | NA | NA |
|  |  | MinE | NA | NA | NA | NA |
|  |  |  |  |  |  |  |
| **SELMA pathway** |  | Cdc48 | GSA120T00008617001 | GSA25T00023867001 | N | within Cdc48 H clade |
|  |  | Cdc48? | GSA120T00019595001 | GSA25T00015438001 | N | similar to no Cdc48 sequences |
|  |  | Cdc48? | GSA120T00010726001 | GSA25T00020284001 | N | similar to no Cdc48 sequences |
|  |  | DERL1 | GSA120T00023603001 | GSA25T00009262001 | N | group with DERL1 H |
|  |  | DERL2 | GSA120T00023194001 | GSA25T00021725001 | N | group with DERL2 H |
|  |  | UFD1 | GSA120T00010479001 | GSA25T00017812001 | N | heterotroph clade? |

Table S4. Number of the different types of introns identified in A25 and A120 genomes.

Percentage of validated canonical and non-canonical introns, classify by their splicing sites motifs. Calculation are based on supported RNA-seq introns (coverage >3), namely 55,290 and 66,565 (onto 81,610 and 90,882 total introns) for A25 and A120, respectively.

|  | **A25** | **A120** |
| --- | --- | --- |
| **Canonical introns GT-AG (Total %)** | **40%** | **35%** |
| **Non-canonical introns (Total %)** | **60%** | **65%** |
| TT-AA (predominant splicing site in A25) | 5 | ND |
| TT-CC (predominant splicing site in A25) | 4 | ND |
| TT-TC (predominant splicing site in A25) | 4 | ND |
| TT-AG (predominant splicing site in A25) | 3 | ND |
| TT-AC (predominant splicing site in A25) | 3 | ND |
| TT-AT (predominant splicing site in A25) | 3 | ND |
| TT-TT (predominant splicing site in A25) | 3 | ND |
| TT-CT (predominant splicing site in A25) | 3 | ND |
| TT-TG (predominant splicing site in A25) | 3 | ND |
| GT-AC (predominant splicing site in A120) | ND | 3 |
| CT-AG (predominant splicing site in A120) | ND | 3 |
| AT-AG (predominant splicing site in A120) | ND | 2 |
| GT-CG (predominant splicing site in A120) | ND | 2 |
| GT-AA (predominant splicing site in A120) | ND | 2 |
| CT-AC (predominant splicing site in A120) | ND | 2 |
| AT-AT (predominant splicing site in A120) | ND | 2 |
| AT-AC (predominant splicing site in A120) | ND | 2 |
| TT-AG (predominant splicing site in A120) | ND | 2 |
| GC-AG (other motif find in dinoflagellates) | 0.2% | 1% |
| GA-AG (other motif find in dinoflagellates) | 0.2% | 1.5% |
| AT-AC (other motif find in dinoflagellates) | 0.9% | 1.5% |
| GG-AG (other motif find in dinoflagellates) | 0.04% | 0.8% |
| GT-TG (other motif find in dinoflagellates) | 0.5% | 1.3% |
| GT-CG (other motif find in dinoflagellates) | 0.3% | 2.1% |
| CT-AG (other motif find in dinoflagellates) | 0.3% | 2.6% |
|  |  |  |

Table S5. Search for RNA editing in A25 and A120 introns

|  | **A25** | **A120** |
| --- | --- | --- |
| Total number of positions with possible RNA editing | 19380 | 21242 |
| Number of positions in introns not in repeated elements (% of the total positions) | 5912 (30%) | 7097 (33%) |
| Number of introns having at least one possible RNA editing (% of the total introns) | 3982 (5%) | 5673 (6.5%) |
| Number of positions found in the first or last 10 bases of the introns (% of the total positions) | 2321 (12%) | 4863 (23%) |
| Number of introns having RNA editing in the first and last 10 bases of the introns (% of the total introns) | 1738 (2%) | 3696 (4%) |
| Number of positions found in the first or last 2 bases of the introns (% of the total positions) | 1578 (8%) | 3085 (14%) |
| Number of introns having RNA editing in the first and last 2 bases of the introns (% of the total introns) | 1270 (1.5%) | 2643 (3%) |

Table S6. Putative *Amoebophrya* A25 and A120 snRNP homologs

*Amoebophrya* snRNPs identified by reciprocal hits analysis, and mcl gene family groups within *H. sapiens*, *P. falciparum* and *T. gondii*. ND corresponds to “not detected”

| **Complex subunit** | ***H. sapiens* (NP)** | ***T. gondii* (TGME49)** | ***P. falciparum* (PF3D7)** | **A25** | **A120** |
| --- | --- | --- | --- | --- | --- |
| **snRNP core (stability and function of U1, U2, U4 and U5 snRNPs)** | | | | | |
| SNRPB | _937859 | _300280 | _1414800 | GSA25T00009626001 | GSA120T00015799001 |
| SNRPD1 | _008869 | _267350 | _1125500 | GSA25T00020775001 | GSA120T00006136001 |
| SNRPD2 | _004588 | _270830 | _0218500 | GSA25T00005714001 | GSA120T00007224001 |
| SNRPD3 | _004166 | _309740 | _0909800 | GSA25T00025008001 | GSA120T00003650001 |
| SNRPE | _003085 | _275750 | _1350200 | GSA25T00019804001 | GSA120T00006897001 |
| SNRPF | _003086 | _213410 | _1126900 | GSA25T00025362001 | GSA120T00013291001 |
| SNRPG | _003087 | _314790 | _0822300 | GSA25T00001657001 | GSA120T00009095001 |
| **U1 snRNP** | | | | | |
| U1-70K | _003080 | _205180 | _1367100 | GSA25T00019007001 | GSA120T00005676001 |
| U1A | _004587 | _309800 | _1306900 | GSA25T00018322001 | GSA120T00025754001 |
| U1C | _003084 | _306380 | _0812700 | GSA25T00015584001 | GSA120T00018889001 |
| PRP40 (A/B) | _001026868 | _306220 | _1316500 | GSA25T00014867001 | GSA120T00025896001 |
| RBM25 | _067062 | _270770 | _0610200 | GSA25T00026804001 GSA25T00021865001 | GSA120T00003034001 GSA120T00002189001 |
| DDX5 | _004387 | _236650 | _1445900 | GSA25T00013401001 | GSA120T00022090001 |
| CA150 | _001035095 | _316180 | _1111200 | GSA25T00009802001 | GSA120T00004838001 |
| **U4/U6 snRNP** | | | | | |
| PRP3 | _004689 | _219790 | _1309300 | GSA25T00014061001 | GSA120T00002339001 |
| PRP4 | _001231855 | _243540 | _1343900 | GSA25T00019043001 | GSA120T00005896001 |
| CypH | _006338 | _230520  _205700  _285760 | _0322000  _0804800  _1115600 | GSA25T00019816001 GSA25T00027189001 GSA25T00006816001 | GSA120T00006823001 GSA120T00000182001 |
| PRP31 | _056444 | _244100 | _0409100 | GSA25T00008446001 | GSA120T00000517001 |
| Snu113 | _004999 | _236580 | _1123900 | GSA25T00018752001 | GSA120T00010172001 |
| snRNP27 | _006848 | _264010 | _0818000 | ND | ND |
| Sad1 | _006581 | _294360 | _1317000 | GSA25T00023451001 | GSA120T00014855001 |
| Snu66 | _005137 | _318140 | _0323700 | GSA25T00016665001 | GSA120T00018941001 |
| Snu23 | _653324 | _275310 | _1243100 | GSA25T00019720001 | GSA120T00006991001 |
| PRP38A | _060531 | _266030 | _1132600 | GSA25T00023879001 | GSA120T00001068001 |
| PRP38B | _116253 | _285230 | _1407300 | GSA25T00022798001 GSA25T00014687001 | GSA120T00014867001 GSA120T00000117001 GSA120T00001396001 |
| **U2 snRNP** | | | | | |
| U2A | _003081 | _229210 | _1369700 | GSA25T00003636001 | GSA120T00010616001 |
| U2B | _003083 | _209690 | _0935000 | GSA25T00010392001 | GSA120T00009931001 |
| SF1 | _004621 | _314860 | _0623600 | GSA25T00002046001 | GSA120T00017418001 GSA120T00012361001 |
| SF3A1 | _001005409 | _246500 | _1474500 | GSA25T00001539001 | GSA120T00009218001 |
| SF3A2 | _009096 | _228000 | _0619900 | GSA25T00018040001 | GSA120T00015561001 |
| SF3A3 | _006793 | _221950 | _0924700 | GSA25T00013095001 | GSA120T00002021001 |
| SF3B1 | _001005526 | _205010 | _0308900 | GSA25T00012209001 | GSA120T00005385001 |
| SF3B2 | _006833 | _314740 | _1461600 | GSA25T00016286001 | GSA120T00002421001 |
| SF3B3 | _036558 | _230960 | _1234800 | GSA25T00020920001 | GSA120T00022307001 |
| SF3B4 | _005841 | _224580 | _1420000 | GSA25T00026577001 | GSA120T00005800001 |
| SF3B5 | _116147 | _248250 | _1018500 | GSA25T00021783001 | GSA120T00001050001 |
| SFB125 | _031398 | ND | ND | GSA25T00011042001 | GSA120T00022140001 |
| SFB14 | _057131 | _305010 | _1224900 | GSA25T00009327001 | GSA120T00014493001 |
| U2AF65 | _001020374 | _234520 | _1468800 | GSA25T00025555001 | GSA120T00005469001 |
| U2AF35 | _001020374 | _236910 | _1119300 | GSA25T00001003001 | GSA120T00008087001 |
| PUF60 | _001258027 | _224850 | _1224300 | GSA25T00018129001 | GSA120T00007081001 |
| SPF30 | _005862 | _286440 | _0323500 | GSA25T00020066001 | GSA120T00024076001 |
| SPF45 | _116294 | _214820 | _1454000 | GSA25T00001210001 | GSA120T00008338001 |
| CHERP | _006378 | _321560 | none | GSA25T00008586001 | GSA120T00012641001 |
| SR140 | _001073884 | _240710 | _1402700 | GSA25T00011628001 | GSA120T00003030001 |
| PRP43 | _001349 | _233520 _312280 _263650 | _0917600 _1030100 | GSA25T00010117001 GSA25T00027084001 GSA25T00000348001 | GSA120T00001099001 GSA120T00013085001 GSA120T00018299001 |
| **U5 snRNP** | | | | | |
| DDX23/PRP28 | _004809 | _298020 | _0518500 | GSA25T00013599001 | GSA120T00011443001 |
| CD2BP2 | _006101 | ND | _1031600 | ND | ND |
| Snu114 | _004238 | _205470 _286080 | _1003800 _1451100 | GSA25T00020040001  GSA25T00003575001 | GSA120T00024060001 GSA120T00010700001 |
| Brr2 | _054733 | _249810 _233390 | _1439100 _0422500 | GSA25T00014432001 GSA25T00003083001 | GSA120T00004033001 GSA120T00026232001 GSA120T00018887001 |
| PRP6 | _036601 | _205220 | _1110200 | GSA25T00000514001 | GSA120T00011220001 |
| PRP8 | _006436 | _231970 | _0405400 | GSA25T00001593001 | GSA120T00009175001 |
| PRP8BP | _004805 | _310860 | _0822800 | GSA25T00000528001 | GSA120T00011213001 |
| DIB1 | _006692 | _270140 | _1231500 | GSA25T00001939001 | GSA120T00017514001 |
| **U6 core (stability and function of U6 snRNP)** | | | | | |
| LSM2 | _067000 | _297140 | _0520300 | GSA25T00014305001 | GSA120T00011592001 |
| LSM3 | _055278 | _298970 | _0819900 | GSA25T00020303001 GSA25T00005714001 | GSA120T00007224001 |
| LSM4 | _036453 | _278950 | _1107000 | GSA25T00020504001 GSA25T00027880001 | GSA120T00002193001 |
| LSM5 | _036454 | _247610 | _1443300 | GSA25T00014455001 | GSA120T00004015001 |
| LSM6 | _009011 | _261470 | _1325000 | GSA25T00025362001 | GSA120T00001208001 GSA120T00013291001 |
| LSM7 | _057283 | _286560 | _1209200 | GSA25T00010229001 | GSA120T00018185001 |
| LSM8 | _057284 | _272630 | _0829300 | GSA25T00014265001 | GSA120T00011562001 |
| **hPrp19/CDC5 (specification of U5 and U6 interactions with RNA)** | | | | | |
| PRPF19 | _055317 | _320210 | _0308600 | GSA25T00022601001 GSA25T00004472001 | GSA120T00014925001 GSA120T00003237001 |
| CRNKL1 | _057736 | _269200 | _0403700 | GSA25T00024249001 | GSA120T00014810001 |
| CDC5L | _001244 | _275480 | _1033600 | GSA25T00003897001 | GSA120T00019196001 |
| ISY1 | _065752 | _203870 | _1472000 | GSA25T00017110001 | GSA120T00020760001 |
| BCAS2 | _005863 | _243620 | _0614400 | ND | ND |
| XAB2 | _064581 | _305240 | _1235900 | GSA25T00018347001 | GSA120T00006726001 |
| PLRG1 | _002660 | _218420 | _0302000 | GSA25T00024611001 | GSA120T00013703001 |
| SYF2 | _056299 | ND | ND | ND | ND |
| SNW1 | _036377 | _233190 | _0218700 | GSA25T00013244001 | GSA120T00023369001 |
| BUD31 | _003901 | _246620 | _0522800 | GSA25T00002733001 | GSA120T00021456001 |
| PPIE | _006103 | ND | ND | GSA25T00006816001  GSA25T00027189001 | GSA120T00000182001 |
| CCDC12 | _001264003 | _279430 | _1451500 | GSA25T00002423001 | GSA120T00023334001 |
| AQR | _055506 | _314410 | _1352700 | GSA25T00019323001 | GSA120T00000623001 |
| CWC15 | _057487 | _270740 | _0722500 | GSA25T00005543001 | GSA120T00017667001 |
| PPIL1 | _057143 | _270560 | _0528700 | GSA25T00021726001 | GSA120T00023192001 |
| **Non-snRNP factors (second step factors) (RNA release)** | | | | | |
| DHX16 | _003578 | ND | _1030100 | GSA25T00027084001 GSA25T00000348001 | GSA120T00013085001 GSA120T00001099001 |
| DDX39B | _004631.1 | ND | _0209800 | GSA25T00012721001 | GSA120T00015321001 |
| DDX46 | _001287789 | ND | _0508700 | GSA25T00024018001 | GSA120T00001622001 |
| SLU7 | _006416 | ND | _0610100 | GSA25T00002586001 | GSA120T00016851001 |
| DHX38 (PRP16) | _054722.2 | ND | _1364300 | GSA25T00008585001 | GSA120T00006589001 |
| CDC40 | _056975.1 | ND | _1220100 | GSA25T00020557001 | GSA120T00002225001 |
| PRPF18 | _003666 | ND | _0922700 | GSA25T00000726001 | GSA120T00006464001 |
| **SR and hnRNP family** | | | | | |
| SRSF1 | _008855 | ND | _0517300 | GSA25T00003447001 GSA25T00003651001 | GSA120T00017256001 |
| PTBP2 | _067013 | ND | _0606500 | ND | ND |
| SRSF4 | _005617 | ND | _1022400 | GSA25T00008688001 | GSA120T00022021001 GSA120T00015640001 |
| hnRNP A | _006796 | _264610 | _0916700 | GSA25T00013062001 | GSA120T00001992001 |
| hnRNP D0 | _112738 | _265530 | ND | GSA25T00016342001 | GSA120T00021290001 |
| hnRNP H | _005511 | _236540 | ND | GSA25T00019779001 | GSA120T00006941001 |
| hnRNP M | _112480 | _262620 | _1006800 | GSA25T00022289001 | GSA120T00021834001 |
| hnRNP U | _114032 | _290270 | ND | ND | ND |

Table S7. Classification into families of non-canonical introns in A25 and A120.

Clustering analyses considering non-canonical introns having RNA-seq to validate the introns junctions (using a coverage>3) and length ≤ 1k, classification based on the IR sequence and sequence similarity (introners) or MITEs-like elements.

|  | A25 | | A120 | |
| --- | --- | --- | --- | --- |
|  | Introners | MITEs | Introners | MITEs |
| Family number | 252 | 34 | 1,954 | 1,121 |
| Family member | 2,039 | 249 | 29,850 | 13,748 |

Table S8. RNAseq read assembly statistics of *Amoebophrya* A25 and A120 corresponding samples from the different time of infection and to the free-living stage (dinospore only).

| **Time of infection (h)** | **Number of reads (M)** | **Assembled reads (%)** | **Number of contigs** | **Contigs average length (nt)** | **N50 contigs (nt)** |
| --- | --- | --- | --- | --- | --- |
| **A120** | | | | |  |
| **Dinospore only** | 217 | 93 | 44,591 | 2,251 | 4,313 |
| **0h (host only)** | 183 | 90 | 202,829 | 945 | 1,581 |
| **6** | 136 | 90 | 203,593 | 909 | 1,543 |
| **12** | 144 | 87 | 210,414 | 825 | 1,386 |
| **18** | 143 | 89 | 222,810 | 922 | 1,556 |
| **24** | 134 | 89 | 225,120 | 890 | 1,510 |
| **30** | 153 | 90 | 225,189 | 986 | 1,705 |
| **36** | 166 | 92 | 222,213 | 976 | 1,693 |
| **A25** | | | | |  |
| **Dinospore only** | 145 | 95 | 41,322 | 2,419 | 4,801 |
| **0 (host only)** | 144 | 83 | 186,115 | 777 | 1,269 |
| **6** | 131 | 81 | 190,459 | 807 | 1,341 |
| **12** | 151 | 85 | 198,130 | 852 | 1,425 |
| **18** | 131 | 82 | 178,304 | 744 | 1,170 |
| **24** | 158 | 86 | 200,408 | 843 | 1,356 |
| **30** | 155 | 89 | 228,002 | 871 | 1,461 |
| **36** | 157 | 91 | 239,274 | 937 | 1,594 |
| **42** | 157 | 91 | 234,415 | 995 | 1,685 |
| **44** | 144 | 91 | 228,678 | 964 | 1,617 |

Table S9. Total number of contigs belonging to samples from different stages of infection and the proportion of them that were aligned against the genomes of both *Amoebophrya* A25 and A120. ND corresponds to “not determined” when no measurement was done.

| **Time of infection (h)** | **Number of contigs** | | **Number of aligned contigs (%)** | |
| --- | --- | --- | --- | --- |
|  | A25 | A120 | A25 | A120 |
| **Dinospore stage** | 41,322 | 44.591 | 37,239 (90%) | 41,810 (94%) |
| **0h (host only)** | 186,115 | 202,829 | 0 | 82 (0,04%) |
| **6** | 190,459 | 203,593 | 8,088 (4%) | 11,592 (5,7%) |
| **12** | 198,130 | 210,414 | 7,740 (3,9%) | 14,781 (7%) |
| **18** | 178,304 | 222,810 | 10,860 (6%) | 29,270 (13%) |
| **24** | 200,408 | 225,120 | 17,489 (8,7%) | 38,252 (17%) |
| **30** | 228,002 | 225,189 | 31,668 (14%) | 44,571 (19,8%) |
| **36** | 239,274 | 222,213 | 39,368 (16%) | 46,519 (21%) |
| **42** | 234,415 | ND | 41,240 (17,6%) | ND |
| **44** | 228,678 | ND | 37,271 (16,3%) | ND |

**Table S10. Metabolic pathway screened in A25 and A120 proteomes.**

D: Detected, ND: Not detected.

| **Number of genes considered** | **Metabolic pathways** | **Functional role** | ***A25*** | ***A120*** |
| --- | --- | --- | --- | --- |
| 4 | Electron transport chain | Conventional Complex I pathway | ND | ND |
| 1 | Electron transport chain | Alternative Complex I pathway | D | D |
| 6 | Electron transport chain | Conventional Complex II pathway | D | D |
| 5 | Electron transport chain | Conventional Complex III pathway | ND | ND |
| 1 | Electron transport chain | Alternative complex III AOX | D | D |
| 9 | Electron transport chain | Conventional Complex IV pathway | Partial cox1 (NUMTS ?) & cox3 is missing | Partial cox1 (NUMTS ?) & cox3 is missing |
| 7 | Electron transport chain | complex V | D | D |
| 1 | Photosynthesis | Cytochrome b6/f complex | ND | ND |
| 4 | Photosynthesis | Photosynthetic electron transport | ND | ND |
| 1 | Photosynthesis | Photosystem-I | ND | ND |
| 3 | Photosynthesis | Photosystem-II | ND | ND |
| 3 | Chlorophyll synthesis | Protoporphyrin IX ---> Chlorophyll a/b | ND | ND |
| 10 | Biosynthesis of Fe-S proteins | Biosynthesis of Fe-S proteins | ND | ND |
| 10 | Pentose cycle and Photosynthetic Carbon Fixation | Reductive pentose (Calvin-Benson cycle) | ND | ND |
| 1 | NADPH production in plastid | Ferredoxin—NADP(+) reductase | ND | ND |
| 2 | Starch synthesis | Floridean starch synthesis | ND | ND |
| 1 | Starch hydrolysis | Floridean starch degradation | ND | ND |
| 2 | Galactose metabolism | synthesis of galactose from UDP-glucose | D | D |
| 3 | Galactose metabolism | D-galactose metabolism | D | D |
| 1 | Trehalose metabolism | Trehalose synthesis from UDP-glucose | D | D |
| 2 | UDP-xylose synthesis |  | D | D |
| 3 | Mannose and fructose metabolism | D-Fructose to GDP-D-Mannose | 2.7.7.13 is missing | 2.7.7.13 is missing |
| 2 | Mannose and fructose metabolism | GDP-D-Mannose to GDP-L-Fucose | D | D |
| 8 | Glycolysis | Glycolysis/gluconeogenesis | D | D |
| 2 | Glycolysis | Direct use of glucose | D: 2.7.1.1 & 2.7.1.2 | D: 2.7.1.1 & 2.7.1.2 |
| 4 | Pentose phosphate cycle | Oxidative phase (NADPH/H+ generation) | ND | ND |
| 3 | Pentose phosphate cycle | Pentose phosphate cycle: non-oxidative phase; ribose-5-phosphate synthesis | D: 2.7.1.15 & 3.6.1.13 | D: 2.7.1.15 & 3.6.1.13 |
| 1 | Pentose phosphate cycle | PRPP generation | D | D |
| 3 | Pentose phosphate cycle | Erythrose-4-phosphate generation: essential substrate in shikimate synthesis | D | D |
| 1 | Pyruvate metabolism | pyruvate to acetyl-CoA: Euglena like Pyruvate dehydrogenase (NADP(+)) | ND | ND |
| 4 | Pyruvate metabolism | pyruvate dehydrogenase complex (PDHC) | E1 is missing | E1 is missing |
| 4 | Pyruvate metabolism | BCKDH complex (see Danne et al. 2013) | ND | ND |
| 1 | Pyruvate metabolism | Anaplerotic reactions, interconversion between malate-pyruvate-oxaloacetate (as in *Cryptosporidium*) | D | D |
| 1 | Pyruvate metabolism | Anaplerotic reactions, PEP to oxaloacetate: EC:4.1.1.31 | ND | ND |
| 1 | Pyruvate metabolism | Anaplerotic reactions, PEP to oxaloacetate: EC:4.1.1.49 | D | D |
| 1 | Pyruvate metabolism | Anaplerotic reaction: pyruvate to Oxaloacetate: EC:6.4.1.1 | D | D |
| 12 | TCA cycle | TCA cycle | All dehydrogenase complexes are missing | All dehydrogenase complexes are missing |
| 2 | TCA bypass | Oxoglutarate bypass= Interconversion between 2-oxoglutarate and succinate as in *Synechococcus* | 4.1.1.71 is missing | D |
| 4 | TCA bypass | GABA shunt=2-oxoglutarate to succinate | 4.1.1.15 is missing | 4.1.1.15 is missing |
| 6 | Peroxisome | PEX core genes (PEX1, 5, 7, 11, 12, and 16) | D | D |
| 2 | Peroxisome | Glyoxylate cycle | ND | D |
| 1 | Amino acid synthesis | Alanine synthesis from pyruvate | D | D |
| 1 | Amino acid synthesis | Interconversion between asparagine and aspartate | ND | D |
| 6 | Amino acid synthesis | Aspartate from histidine | ND | ND |
| 1 | Amino acid synthesis | Oxaloacetate to aspartate interconversion | D | D |
| 5 | Amino acid synthesis | Glutamate from arginine | ND | ND |
| 1 | Amino acid synthesis | Glutamate synthesis from 2-oxoglutarate | D | D |
| 1 | Amino acid synthesis | Glutamine from glutamate | D | D |
| 5 | Amino acid synthesis | Threonine synthesis from aspartate | D | D |
| 3 | Amino acid synthesis | Serine synthesis from 3P-glycerate | D | D |
| 1 | Amino acid synthesis | Glycine synthesis from serine + Tetrahydrofolate | D | D |
| 1 | Amino acid synthesis | Glycine synthesis from serine + Glyoxylate | D | D |
| 5 | Amino acid synthesis | Glycine synthesis from choline | ND | ND |
| 1 | Amino acid metabolism | Glycine - threonine interconversion | D | D |
| 3 | Amino acid metabolism | Glycine cleavage system | D | D |
| 2 | Amino acid synthesis | Cysteine synthesis from serine (as Symbiodinium kaw.) | D | D |
| 2 | Amino acid synthesis | Cysteine synthesis from serine (as human) | D | D |
| 9 | Amino acid synthesis | Histidine synthesis | ND | ND |
| 6 | Amino acid synthesis | Isoleucine, valine & leucine synthesis | ND | ND |
| 6 | Amino acid degradation | Degradation of Branched chain amino acid (Isoleucine, valine & leucine) (complementary to BCKDH complex) | BCKDH & 4.1.3.4 is missing | BCKDH & 4.1.3.4 is missing |
| 5 | Amino acid synthesis | Lysine synthesis from aspartate | D | 4.3.3.7 & 4.1.1.20 are missing |
| 5 | Amino acid degradation | Lysine degradation: lysine to Glutaryl-Coa | ND | ND |
| 3 | Amino acid degradation | Lysine degradation: Glutaryl-CoA to Acetyl CoA | D | D |
| 6 | Amino acid synthesis | Methionine synthesis | ND | 2.3.1.31 is missing |
| 8 | Amino acid metabolism | Methionine salvage SAM ---> S-methyl-thioadenosine ---> Methionine | ND | ND |
| 4 | Amino acid metabolism | L-methionine degradation to L-homocysteine | D | D |
| 1 | Amino acid metabolism | Polyamine synthesis: Putriscine synthesis from ornithine | D | ND |
| 1 | Amino acid metabolism | Polyamine synthesis: spermine & spermidine synthesis | D | D |
| 3 | Amino acid synthesis | Shikimate pathway (produce chorismate) | ND | 4.2.3.5 may be fused with AROM |
| 5 | Amino acid synthesis | Phenylalanine & tyrosine synthesis | ND | ND |
| 1 | Amino acid synthesis | Tyrosine synthesis from Phenylalanine | D | D |
| 5 | Amino acid synthesis | Tryptophan synthesis from chorismate | ND | D |
| 10 | Amino acid synthesis | arginine synthesis: Glutamate to ornithine & urea cycle | ND | ND |
| 3 | Amino acid metabolism | Proline synthesis from glutamate as plants | D | D |
| 1 | Amino acid metabolism | Ornithine synthesis | D | D |
| 1 | Amino acid metabolism | Proline hydroxylation | D | D |
| 5 | Amino acid metabolism | Selenocompoud metabolism selenocysteine to protein | D | D |
| 3 | Amino acid metabolism | Selenocompoud metabolism: selenocysteine to selenomethionine | D | D |
| 3 | Fatty acid synthesis | Fatty acid elongation in cytosol (FAS I) | D | D |
| >6 | Fatty acid synthesis | Fatty acid synthesis in the apicoplast/plastid (FAS II) | ND | ND |
| 4 | Fatty acid synthesis | Fatty acid elongation in ER (Elongase pathway) | D | D |
| 5 | Fatty acid recycling and degradation | beta-oxidation of fatty acid | D | D |
| 6 | Glycerolipid metabolism | Triacylglycerol synthesis from glycerol OR recylcing of phospholipids | D | D |
| 3 | Sterol | Dolichol salvage & synthesis | D | D |
| 2 | Thylakoid lipid synthesis (2 possible routes) | Galactosyl-diacyl glycerol synthesis | ND | ND |
| 2 | Thylakoid lipid synthesis (2 possible routes) | Sulfo-quinovosyl-diacyl glycerol synthesis | ND | ND |
| 6 | Isoprenoid (isopentenyl diphosphate (IPP)) biosynthesis | Mevalonic acid pathway | ND | ND |
| 6 | Isoprenoid (isopentenyl diphosphate (IPP)) biosynthesis | MEP/DOXP pathway | ND | ND |
| 2 | Steroid Metabolism | squalene 2,3-epoxide synthesis from IPP | ND | ND |
| 11 | Steroid Metabolism | Cholesterol ester synthesis from squalene 2,3-epoxide | D: 6 proteins | D: 6 proteins |
| 4 | Steroid Metabolism | Methylene-lophenol from squalene 2,3-epoxide | ND | ND |
| 9 | Purine metabolism | IMP production from PRPP | D | D |
| 2 | Purine metabolism | 3',5' cyclic AMP/GMP | D | D |
| 3 | Purine metabolism | nucleoside catabolism | D | D |
| 2 | Purine salvage | Purine salvage from adenosine (EC: 2.7.1.20) | D | D |
| 4 | Purine salvage | Purine salvage from adenine, inosine, hypoxanthine | D: 3 proteins | D: 3 proteins |
|  | Purine salvage | nucleoside transporter | D | D |
| 6 | Pyrimidine metabolism | UMP synthesis from Glutamine | D | D |
| 1 | Pyrimidine salvage | from uridine & uracil & cytidine (EC: 2.7.1.48 2.4.2.9) | D | D |
| 1 | Pyrimidine salvage | from thymidine (EC: 2.7.1.21) | D | D |
| 8 | Folate metabolism (Vitamin B9) | Vitamin B9 (Folate) synthesis from GTP | D : 5 proteins | D : 5 proteins |
| 6 | Folate metabolism (Vitamin B9) | Recycling of folate (one carbon pool by folate) | D : 5 proteins | D : 5 proteins |
| 6 | Folate metabolism (Vitamin B9) | Molybdopterin cofactor synthesis from GTP | ND | ND |
|  | Folate metabolism (Vitamin B9) | Vitamin B9 (Folate) synthesis from chorismate | ND | ND |
| 3 | Co-factor Metabolism | Lipoic acid synthesis (in apicoplast for apicomplexan) | ND | ND |
| 3 | Co-factor Metabolism | Lipoic acid metabolism: salvage in the mitochondrion | D | D |
| 1+7 | Co-factor Metabolism | Heme synthesis | ND | HemH and HemF/N are missing |
| 6 | Co-factor Metabolism | Ubiquinone-n synthesis from chorismate | ND | ND |
| 3 | Co-factor Metabolism | Nicotinate and nicotinamide metabolism from tryptophan/aspartate | ND | ND |
| 4 | Co-factor Metabolism | Nicotinate ribonucleotide ---> NAD+ <---> NADP+ | D | D |
| 2 | Co-factor Metabolism | salvage of nicotinate (vitamin B3) and nicotinamide | D | D |
| 6 | Pantothenate and CoA biosynthesis | Pantothenate synthesis from pyruvate (EC:2.2.1.6)/valine (EC:2.6.1.42) | D: 2.6.1.42, 6.3.2.1 is missing | D: 2.2.1.6 & 2.6.1.42, 6.3.2.1 is missing |
| 5 | Pantothenate and CoA biosynthesis | CoA synthesis from pantothenate | D | 4.1.1.36 is missing |
| 2 | Co-factor Metabolism | Vitamin B6 (Pyridoxal phosphate) de novo synthesis: DOXP-independent route | D | D |
| 3 | Co-factor Metabolism | Vitamin B6 (Pyridoxal phosphate): phosphorylation of salvaged pyridoxal | D | D |
| 6 | Co-factor Metabolism | Riboflavin (Vitamin B2) synthesis | 3.1.3.104 is missing | D |
| 3 | Co-factor Metabolism | Riboflavin (Vitamin B2) to FAD | D | D |
| 3 | Co-factor Metabolism | Thiamin (vitamine B1) biosynthesis | ND | ND |
| 3 | Co-factor Metabolism | Thiamine to thiamine pyrophosphate | D | D |
| 1 | Nuclear organization | DVNP | D | D |
